# Phage Predation Shapes the Population Structure of Shiga-Toxigenic *Escherichia coli* O157:H7 in the UK: An Evolutionary Perspective

**DOI:** 10.3389/fgene.2019.00763

**Published:** 2019-08-30

**Authors:** Lauren A. Cowley, Timothy J. Dallman, Claire Jenkins, Samuel K. Sheppard

**Affiliations:** ^1^The Milner Centre for Evolution, University of Bath, Bath, United Kingdom; ^2^National Infection Services, Public Health England, London, United Kingdom

**Keywords:** phage, *Escherichia coli* O157:H7, population structure, host, evolution

## Abstract

Bacterial–host interactions are non-linear and actually threefold, involving significant selection through predatory lytic bacteriophages in the host environment. In studies of human and animal gut microbiome bacteria, it is important to consider phage in all host–pathogen interactions. We use an important zoonotic pathogen, Shiga toxigenic *Escherichia coli* (STEC) O157:H7, to investigate this. Our study provides evidence that phage resistance profiles are well maintained at the sub-lineage level with variation in profiles within sub-lineages uncommon. This indicates that phage resistance heterogeneity happened early on in the STEC O157:H7 natural history and that occasional “wobbles” do not often outcompete the stable lineage unless combined with a competitive advantage. We discuss an example of this in the acquisition of *stx2a* that, while an important virulence factor, also conveys increased phage cross-resistance. We also discuss the role of phage resistance in co-occurrence of the three stable lineages worldwide and whether differing phage resistance is maintaining diversity.

## Introduction

Bacteria are ubiquitous colonizers of plant and animal hosts. The increasing availability of large bacterial genome datasets is revealing variation in strains and species that reflect genetic isolation in, and adaptation to, the host (Sheppard et al., 2018). It is tempting to consider the host-associated microbiota as a stable community, but this is not the case. Rather, the observed population sampled from a given host is a snapshot of stochastic mechanisms in an ongoing microbial war in which only the fittest survive to pass their genes onto the next generation. The ecological genomics of host–bacterial interactions is extremely complex with genetic variation in both the host and bacterium contributing to successful colonization and proliferation. Broadly, however, it is useful to consider the extant bacterial population as reflecting the outcome of competition with the host response, with other bacteria and with bacteriophage (viruses that infect bacteria).

Shiga-toxigenic *Escherichia coli* (STEC) serotype O157:H7 is a zoonotic, foodborne pathogen that causes gastrointestinal disease in humans. Despite being relatively rare compared with other bacterial gastrointestinal infections, for example, *Campylobacter* and *Salmonella*, STEC O157:H7 infections are of significant public health concern due to potential disease severity. Symptoms range from abdominal cramps and nausea to severe bloody diarrhea and, in 5–14% of cases, infection leads to hemolytic uremic syndrome (HUS), a severe and potentially fatal condition (Adams et al., 2016). Cattle and other ruminants are natural reservoirs for STEC O157:H7, where the bacterium is part of the commensal microbiota, and transmission to humans occurs through direct or indirect animal contact or through consumption of contaminated food or water (Byrne et al., 2015). Around 20% of the STEC O157:H7 genome is made up of prophage (Hayashi et al., 2001) and the key virulence factor, *stx*, is encoded on lambdoid phages (Scotland et al., 1985). The Shiga toxin (*stx*) can be divided into subtypes *stx1a-1d* and *stx2a-2g* (Scheutz et al., 2012). The *stx2a* subtype is associated with adverse clinical outcomes, including progression to HUS (Dallman et al., 2015).

There are three major lineages of STEC O157:H7, lineage I, II, and I/II. The STEC O157:H7 clone is globally disseminated but analysis of the population structure reveals evidence of the expansion of certain lineages and/or sub-lineages in different geographical regions periodically during its evolutionary history (Dallman et al., 2015). When STEC O157:H7 emerged in the UK in the 1980s, the dominant lineage associated with causing gastrointestinal disease in humans was I/II (Dallman et al., 2015; Adams et al., 2016). During the 1990s, a rapid decline in the number of human STEC O157:H7 cases caused by strains belonging to lineage I/ll coincided with an equally rapid increase in cases caused by strains belonging to sub-lineage Ic, and a more gradual increase in cases caused by strains belonging to sub-lineage IIc (Dallman et al., 2015; Adams et al., 2016). Over the last decade, a gradual decline in human cases caused by STEC O157:H7 sub-lineage Ic has coincided with an increasing trend in the number of cases caused by sub-lineage IIb (Byrne et al., 2018). The emergence of sub-lineages I/II, Ic, and IIb as a cause of human disease coincided with the acquisition of *stx2a* integrated into the genome on a prophage.

Bacteriophage display different host (bacterium) specificity with some phage having broad host ranges and others capable of only infecting a limited strain set. This phenomenon is exploited by phage typing, an *in vitro* method where the specificity of different bacteriophages is used to differentiate bacterial strains. The STEC O157:H7 phage typing scheme has been used at Public Health England (PHE) for more than 20 years as an epidemiological tool for the rapid detection and subsequent investigation of outbreaks. Following the implementation of whole genome sequencing at PHE, retrospective studies revealed that each bacterial sub-lineage broadly correlates with certain phage types (PT); lineage I/ll comprises PT2, sub-lineage Ic correlates with PT21/28, and the recent emergence of sub-lineage IIb coincided with a change in the dominant PT associated with this sub-lineage from PT4 or PT1 to PT8 (Dallman et al., 2015; Byrne et al., 2018). The O157:H7 PT scheme is a phenotypic assay that produces a profile of susceptibility and resistance based on 16 typing phages, 14 T4-like lytic coliphages and 2 T7-like lytic coliphages. The results of phage typing can be condensed into susceptibility (defined as any kind of lysis with those phages) or resistance (defined as no lysis with those phages) for each of the typing phage similarity groups. These groups have been previously defined as T4 Group 1—TP1, 8, 11, and 15; T4 Group 2—TP3, 6, 7, and 13; T4 Group 3—TP4, 5, and 14; and T7—TP9 and 10 (Cowley et al., 2015).

In the same way that pathogenic bacteria and viruses are in a continual struggle to survive the human immune system of their hosts, bacteriophage are in a simultaneous arms race with bacteria to survive and pass on their genetic information through infection of their bacterial hosts. We propose that this dynamic has made a significant contribution to the population structure and therefore the evolutionary history of STEC O157:H7. Phage predation has posed an evolutionary pressure on this pathotype and certain phage resistance profiles have become fixed in the population. STEC O157:H7 is susceptible to lysogenic phage and is known to exclude some prophage through phage–phage interactions and superinfection immunity (Labrie et al., 2010). Horizontal gene transfer, such as plasmid carriage, and prophage carriage provides resistance mechanisms to lytic phages, thereby conferring a selective advantage to those strains.

The interaction of phage and bacterium within the reservoir host (ruminants) is a key factor in shaping the STEC O157:H7 population to which humans are exposed. However, there are multiple phenotypes that affect the population structure, such as super shedding, colonization in the terminal rectum, and host immunity. The population structure observed in human disease is not the natural ecology where selection takes place and different strain diversity is seen in cattle to people. However, we can use measurements of strains causing disease in humans as markers of prevalence in cows. Strains causing disease in humans also have to be successful in their primary ruminant hosts and can provide indications of the selective environment of the primary host.

Heterogeneity in phage resistance profiles within the population structure of STEC O157:H7 can potentially be linked to short-term changes in accessory genome but can also become fixed as stable lineage associations. Here, by combining information from phage typing profiles (Cowley et al., 2015) and whole genome sequences, we investigate how the fixation of resistance in lineages relates to phenotypes in the rest of the population structure. Observations of bacteria–phage interactions (Cowley et al., 2016), in the context of a reconstructed phylogeny, are then used to understand how bacteriophage resistance and susceptibility can influence the evolutionary history of STEC O157.

## Results and Discussion

### Evidence of Fixed Phage Resistance

Phage resistance and susceptibility in STEC O157:H7 is a complex phenotype involving the interaction of large numbers of genes and gene families (Cowley et al., 2018). Although some phage resistance can be termed “fixed” by its presence in the majority of the clade, some infrequent deviation from the “fixed” phenotype can be observed in the phlyogeny. This may be due to layering of additional mutations in the genome or accessory gene acquisitions on top of a prophage or accessory gene that has caused the “fixed” phenotype.

**Figure 1:**
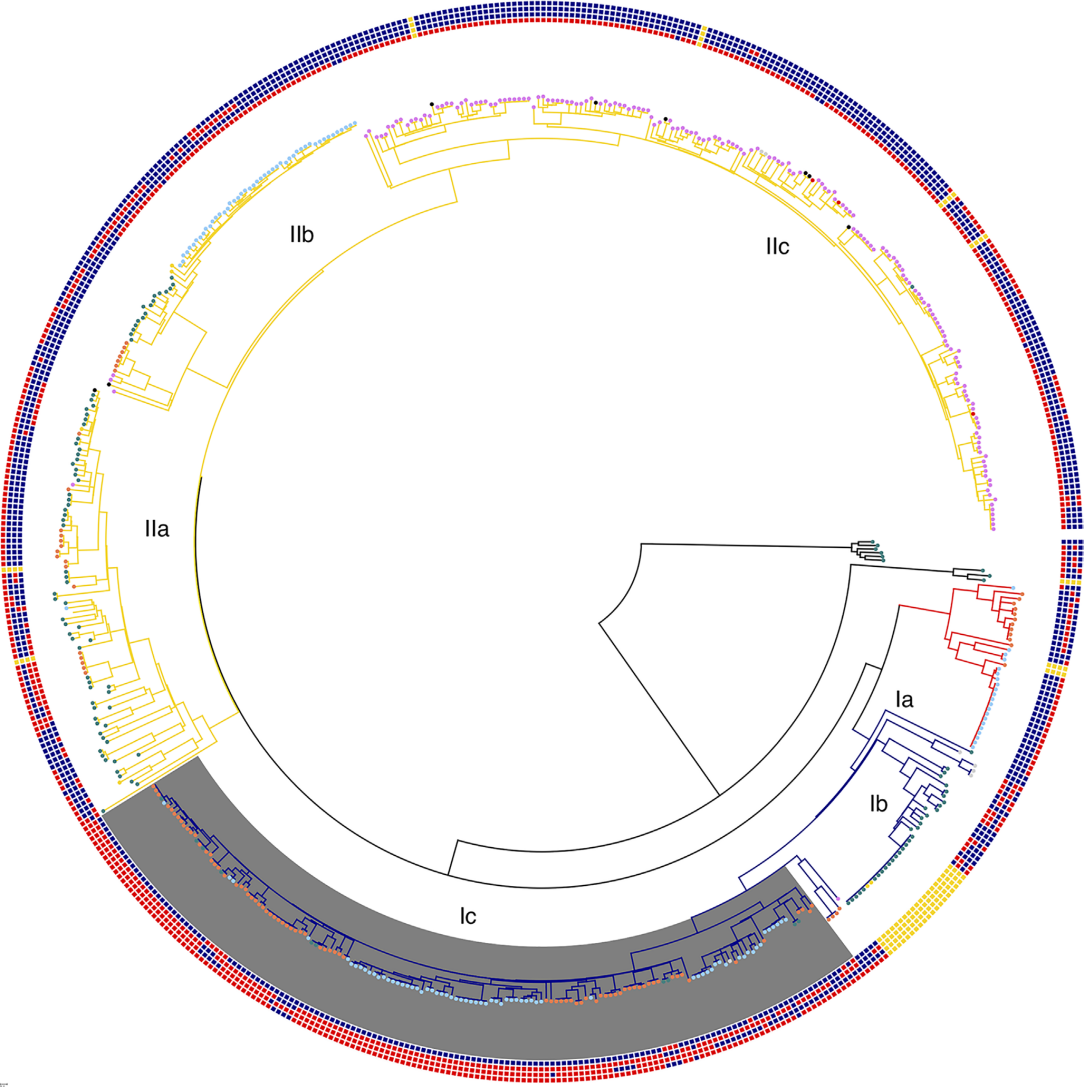
Phylogenetic tree displaying all clinical cases of STEC O157 from the UK in 2017 (*n* = 580) (**Supplementary Table 1**). Clades are labeled by color (clade I—blue, clade I/II—red, clade II—yellow). Phage resistance phenotypes are labeled as colored squares concentrically around the outside in the following order: T7 coliphages, Group 1 T4 coliphages, Group 2 T4 coliphages, and Group 3 T4 coliphages (Cowley et al., 2015). Red signifies resistance, blue signifies susceptibility, and yellow signifies no information. OmpC allele with C at position 921 instead of T highlighted in gray. Nodes are labeled by stx profile. Violet signifies stx2c stx1a, blue signifies stx2a, green signifies stx2c, orange signifies stx2a stx2c, red signifies stx1a, black signifies stx2a stx2c stx1a, and gray signifies stx2a stx1a.

Phage resistance and susceptibility was regarded as fixed when all members of a clade displayed the same phenotype. As seen in **Figure 1**, with the exception of one minor cluster in lineage IIc, Group 1 T4 coliphage susceptibility was fixed in lineages IIb and IIc, and with the exception of five minor clusters, T7 phage resistance was fixed in the most recently emerged clade of lineage IIb and lineage IIc. In contrast, T7 phage susceptibility was seen to be fixed in the most recent clade of lineage Ic, apart from two minor clusters revealed in **Figure 1**.

Within lineages, phage resistance profiles and phylogenetic clades are generally consistent. For this to be the case, there must be phage resistance phenotype fixation within phylogenetic clades. Therefore, it is likely that most phage resistance diversity happened early on in the STEC O157:H7 history. Occasionally we see “wobbles” introduced to the phenotype, but this is unlikely to outcompete other strains unless a competitive advantage is also provided.

### Evidence of Transient or Horizontally Transferred Phage Resistance

Phage resistance and susceptibility were regarded as transient when the phenotype changed within phylogenetic clades within a relatively short evolutionary history. There were five separate evolutionary events within sub-lineage IIc where resistance to the group 3 coliphages has evolved in a background of susceptibility to all but the T7 coliphages (**Figure 1**). This represents a robust example of homoplasy with multiple separate, but presumed identical, evolutionary events (defined as a genomic change happening on a branch that is maintained in all subsequent descendants) leading to the development of resistance to the group 3 T4 coliphages occurring in different ancestral backgrounds within a short evolutionary time frame. The increased resistance to the group 3 T4 coliphages in sub-lineage IIc was not related to the acquisition or loss of the *stx*-encoding prophages and was not associated with any change in *stx*-profile. In one example identified during an outbreak of STEC O157:H7 in the UK in 2013, where the contaminated food vehicle was identified as herbs imported from the Mediterranean region, resistance to the group 3 coliphages was shown to be horizontally transferred on a plasmid (Cowley et al., 2016). Alternative methods of transient horizontal transfer for resistance to this group of T4 coliphages are likely to exist.

Approximately 30 years ago, susceptibility to the group 2 T4 coliphages in sub-lineage Ic was lost following the acquisition of the *stx2a*-encoding prophage (Shaaban et al., 2016). However, as seen in **Figure 1**, within the most recently emerged clade of sub-lineage Ic, there are four evolutionary events associated with the transient return of group 2 T4 coliphages susceptibility. The opposite occurs in lineage II, which is characterized by exhibiting susceptibility to the group 2 T4 coliphages but where minor clusters have developed resistance (1 evolutionary event in IIc, 5 evolutionary events in IIb, and 4 evolutionary events in clade IIa). In certain clusters, this may be linked to the acquisition of *stx2a* encoding bacteriophage, as described above. However, acquisition of *stx2a* does not explain all the changes linked to T4 susceptibility and resistance in lineage II, as in sub-lineage IIb acquisition of *stx2a* is associated with conversion to an increasingly susceptible profile to the T4 coliphages. This could also involve the gain or loss of other elements on the *stx*-phage backbone other than the toxin and it could be different backbone determinants in the two lineages causing these differing phenotypes. Multiple genes have been identified as being involved in the resistance and susceptibility of a member of this group of T4 coliphages (Cowley et al., 2018).

The evolutionary advantage of virulence factors is often questioned in pathogenic bacteria, but *stx2a*, which is commonly associated with severe clinical outcomes like HUS, has also been shown to convey increased cross-resistance to phage (Shaaban et al., 2016) and therefore a potential survival advantage to those strains carrying the *stx2a*-encoding prophage. It has also been suggested that *stx* gene carriage plays a role in the ecological niche through partial induction of the toxic genes at a low enough frequency that it becomes a positive selective force in toxin-dependent killing of eukaryotic cells (Los et al., 2013). Phage resistance in general is regarded as a survival advantage, but at present, little is known about the cost of phage resistance compared to what is known about the fitness cost of antibiotic resistance (Melnyk et al., 2015). Furthermore, it is not yet understood whether phage resistance is often transient because it is most commonly found on selectable mobile elements like plasmids or prophage or whether chromosomal variation is actively selected just as commonly.

### OmpC Mutation

The most common and well-studied mechanism of phage resistance is through adaptation of outer membrane proteins that act as receptors for tailed bacteriophage. This is a mechanism that has been studied in detail since the early days of molecular and evolutionary biology (Luria and Delbrück, 1943; Demerec and Fano, 1945). OmpC is a known T4 phage receptor, so mutations in it could be associated with phage susceptibility changes. As indicated in **Figure 1**, clade Ic has gained a point mutation in outer membrane protein C that is absent from all other lineages. This is coupled with a marked increase in phage resistance in clade Ic. Further investigation of the point mutation revealed that clade Ic isolates had mutated from T to C at position 921 of *ompC* (Strain 9000 GenBank protein ID API32766.1). However, the point mutation is a synonymous mutation, so it does not change the amino acid coding sequence. This decreases the likelihood of an interference of phage receptor binding through protein folding and conformational changes but may play a role in internal cell messaging or regulation of the gene. Exploratory studies into the role of this SNP in phage susceptibility are warranted. T4-like bacteriophage have also been shown to interact with the LPS layer of bacteria as a binding site so further work to investigate mechanisms of resistance should also focus on mutations in LPS proteins.

### Population Shaping

With respect to sub-lineage Ic, the increase in clinical cases was correlated with an increase in resistance to the T4 coliphages compared to more susceptible ancestors. As well as starting to carry a potent virulence factor that will increase severe clinical cases, increased phage resistance is likely to be linked to the success of the lineage as it conveys a distinct evolutionary advantage to strains exposed to niches inhabited by T4 coliphages, such as the ruminant gut. Conversely, strains of STEC O157:H7 belonging to sub-lineage IIc cause a high burden of gastrointestinal disease in humans due to the carriage of *stx1a* that is associated with increased risk of bloody diarrhea but are largely susceptible to all the T4 coliphage in the phage typing scheme. One hypothesis is that sub-lineage IIc occupies a different reservoir or environmental niche to sub-lineage Ic characterized by the lack of a T4 coliphage lytic predator. Strains from both lineages can be isolated from the same cows but are often associated with different outbreak vehicles (Ic are often associated with meat and animal contact and IIb and IIc are often associated with salad and vegetables) (Launders et al., 2016; Mikhail et al., 2017; Sinclair et al., 2017). In this way, the combination of genetic variation in bacterium and phage in a particular host niche leads to heterogeneity among STEC O157:H7 to which humans are exposed.

Our analysis showed that of the STEC O157:H7 sub-lineages currently causing the greatest burden of gastrointestinal disease in the human population in the UK, specifically sub-lineages Ic, IIc, and IIb, sub-lineage Ic displayed cross-resistance to the T4 coliphages but not the T7 coliphages, whereas sub-lineage IIc and the emerging sub-lineage IIb were susceptible to all the T4 coliphages but resistant to the T7 coliphages. Therefore, the dominant lineage I and II sub-lineages have opposing phage resistance profiles with opposite phenotypes for the four phage groups tested. This observation provides some insight into the coexistence of different sub-lineages of STEC O157:H7 in the UK and the need to occupy separate niches—potentially even within the same host rumen. The dominant lineage II clones are likely to succeed in niches where T7-like phages are abundant and the dominant lineage I clone is likely to succeed in T4 coliphage-rich environments.

Alternatively, mechanisms of lytic phage resistance could be under Negative Frequency Dependent Selection (NFDS), which has recently been associated with bacterial accessory gene content (Corander et al., 2017; McNally et al., 2018). NFDS maintains genes at an intermediate frequency within the population and those genes under NFDS are prevented from becoming more common by loss of fitness at high frequency. This would apply to mechanisms of phage resistance that if present in the whole population would become the target of all phage predation strategies. Mechanisms at intermediate frequency distribute phage predation across the population to prevent kill-the-winner dynamics (Rodriguez-Valera et al., 2009). This would maintain opposite phage susceptibility phenotypes in co-occurrence.

## Conclusions

Chromosomal background determinants of phage susceptibility phenotypes are likely to be established and fixed in specific lineages and sub-lineages at the time of divergence, but transient susceptibility and resistance profiles observed in these lineages may be shaped by the current niches that they occupy. This relationship between clone and phage population in environmental niche is likely to be a significant driver in shaping the population structure of bacteria including STEC O157:H7. Further phage susceptibility testing would expand the work described in this perspective. Additional testing of different lytic phages that infect STEC O157:H7 may provide a better understanding on how phage populations in different environmental niches and animal reservoirs influence the evolution and emergence of the STEC O157:H7 sub-lineages causing the greatest burden of gastrointestinal disease. In conclusion, our results have shown that predatory phage effects in the primary ruminant host affect strains causing disease in the secondary human host and may maintain diversity in strains observed in human disease worldwide.

## Materials and Methods

### Whole Genome Alignment and Recombination Removal

Sequencing reads from 580 isolates of STEC O157:H7 from human cases in England and Wales, submitted to the Gastrointestinal Bacterial Reference Unit (GBRU) in 2017, were downloaded from the sequence read archive. The reads were trimmed (Bolger et al., 2014) and mapped using BWA-SW (Li and Durbin, 2010) to the STEC O157:H7reference strain Sakai (GenBank accession BA000007). The alignment map file from BWA was sorted and indexed into a binary alignment map (BAM) with Samtools (Li et al., 2009), and GATK2 (McKenna et al., 2010) was used to produce a Variant Call Format (VCF) file that was parsed to call SNPs of high quality (MQ ≥ 30, DP ≥ 10, GQ ≥ 30, variant ratio ≥ 0.9). Whole genome alignment files were used with Gubbins (Croucher et al., 2015) to identify regions of high SNP density that were assumed to be areas of recombination. These recombination regions were removed in new alignment files and mutations outside of these regions were used to create a phylogeny that better reflect the clonal frame of the population using RAxML (Stamatakis, 2014).

### Phage Resistance Phenotyping

Public Health England (PHE) have used phage typing as a way of broadly discriminating and clustering strains of STEC O157 for more than 20 years. This involves a panel of 16 typing phages (TP) that the strains are assayed against to produce a resistance profile corresponding to phage type (Cowley et al., 2015). Groupings can be used instead of individual phages as, in most cases, strains have the same reaction to all members of the similarity groupings. In those cases where there is heterogeneity of phenotype within the group, the stronger reaction/clearer phenotype has been chosen as representative.

Briefly, STEC O157:H7 strains for typing are flooded on a Difco nutrient agar plate with 1 ml of broth culture. Dried plates are spotted with 10 µl of each of the 16 typing phages following a template and incubated overnight. Profiles of lysis for each of the typing phages are then recorded the next day.

### Stx Prophage Carriage Determination

*Stx* profiling is an important assay in public health surveillance of STEC O157 and is carried out routinely at PHE who provided data for this study. Shiga toxin subtyping is performed through a mapping technique to reference Shiga toxin sequences as previously described (Ashton et al., 2015). Briefly, reads were mapped to *stx1a, stx1c, 1d, 2a, 2b, 2c, 2d, 2e, 2f*, and *2g* sequences (taken from Scheutz et al., 2012) using BWA-MEM (http://bio-bwa.sourceforge.net/). Reads that mapped to more than one place in the reference set (i.e., ambiguous reads) were removed from the resultant SAM file using Samtools (Li et al., 2009). If at least 10 reads and 90% of the total reads concordantly mapped to discriminatory positions for a specific subtype, then a positive match was returned for that subtype.

### OmpC Allele Determination

The OmpC coding region was identified in strain 9000 (GenBank accession CP018252.1, protein ID API32766.1, locus tag BFL16_​16860) (Shaaban et al., 2016). This reference sequence was used to query all the STEC 2017 assemblies using blastn with e-value 0.0000000001. One hundred percent identity matches and coverage were seen for all clade Ic isolates and all other clade isolates had a T at position 921 instead of the C seen in strain 9000.

## Data Availability

The datasets generated for this study can be found in Supplementary table 1, listed in Table S1.

## Author Contributions

LC conceived, designed and carried out the research. TD and CJ provided data. All authors contributed to writing the manuscript.

## Funding

LC is supported under the University of Bath prize fellowship scheme. SS is a principal investigator for the MRC CLIMB consortium (MR/L015080/1). TD and CJ are funded through PHE.

## Conflict of Interest Statement

The authors declare that the research was conducted in the absence of any commercial or financial relationships that could be construed as a potential conflict of interest.
