# Supplementary material for: Phage Predation Shapes the Population Structure of Shiga-Toxigenic Escherichia coli O157:H7 in the UK: An Evolutionary Perspective
Source: Front Genet. 2019 Aug 30;10:763. doi: 10.3389/fgene.2019.00763 (PMC6730009; doi:10.3389/fgene.2019.00763)
Supplement: Supplementary file 1 [file Table_1.docx]

**Supplementary materials**

**Supplementary table 1:** Table detailing the strains used in our study according to Sequence Read Archive (SRA) accession, Phage Type (Cowley et al., 2015), Resistance to 4 groups of phages tested of the T7 and T4 variety, *stx* profile, SNP address (Dallman et al., 2018), sub-lineage (Yokoyama et al., 2016) and outbreak association. Outbreak association data was provided by PHE but has also been published (Treacy et al., 2019, Pereboom et al., 2018).

|  | | | **T4 Group:** | | |  | | | | |
| --- | --- | --- | --- | --- | --- | --- | --- | --- | --- | --- |
| **SRA ACCESSION** | **Phage Type** | **T7** | **1** | **2** | **3** | ***stx* profile** | **SNP ADDRESS** | **Sub-lineage** | **Outbreak** |  |
| SRR6052886 | PT 14 | No | No | No | No | stx2c | 1.1.1239.3058.3800.3999.4571 | Ib |  |  |
| SRR7250961 | PT 14 | No | No | No | No | stx2c | 1.1.1239.3058.3800.3999.4571 | Ib |  |  |
| SRR6052937 | PT 8 | Yes | No | No | No | stx2c | 1.1.1558.2978.3664.3841.4360 | Ib |  |  |
| SRR6053016 | PT 31 | Yes | Yes | No | Yes | stx2c | 1.1.1630.3078.3828.4035.4614 | Ib |  |  |
| SRR6052872 | PT 8 | Yes | No | No | No | stx2c | 1.1.1638.3096.3853.4061.4651 | Ib |  |  |
| SRR7244359 | PT 14 | No | No | No | No | stx2c | 1.1.1660.3133.3927.4143.4756 | Ib |  |  |
| SRR7277753 | PT 14 | No | No | No | No | stx2c | 1.1.1721.3244.4099.4342.5018 | Ib |  |  |
| SRR6052933 | PT 32 | No | Yes | No | Yes | stx2c | 1.1.736.2853.3857.4066.4658 | Ib |  |  |
| SRR6053005 | PT 31 | Yes | Yes | No | Yes | stx2c | 1.1086.1590.3038.3772.3969.4535 | Ib |  |  |
| SRR6053033 | PT 14 | No | No | No | No | stx2c | 1.1092.1628.3075.3824.4030.4608 | Ib |  |  |
| SRR7277726 | PT 31 | Yes | Yes | No | Yes | stx2c | 1.1098.1654.3120.3901.4113.4715 | Ib |  |  |
| SRR5461667 | RDNC |  |  |  |  | stx2c | 1.727.1041.2979.3665.3842.4363 | Ib | OUTBREAK |  |
| SRR6001299 | RDNC |  |  |  |  | stx2c | 1.727.1041.2979.3665.3842.4363 | Ib | OUTBREAK |  |
| SRR6052730 | RDNC |  |  |  |  | stx2c | 1.727.1041.2979.3665.3842.4363 | Ib | OUTBREAK |  |
| SRR6052757 | RDNC |  |  |  |  | stx2c | 1.727.1041.2979.3665.3842.4363 | Ib | OUTBREAK |  |
| SRR6052774 | RDNC |  |  |  |  | stx2c | 1.727.1041.2979.3665.3842.4363 | Ib | OUTBREAK |  |
| SRR6052782 | RDNC |  |  |  |  |  | 1.727.1041.2979.3665.3842.4363 | Ib | OUTBREAK |  |
| SRR6052829 | RDNC |  |  |  |  | stx2c | 1.727.1041.2979.3665.3842.4363 | Ib | OUTBREAK |  |
| SRR6052867 | RDNC |  |  |  |  | stx2c | 1.727.1041.2979.3665.3842.4363 | Ib | OUTBREAK |  |
| SRR6053027 | RDNC |  |  |  |  | stx2c | 1.727.1041.2979.3665.3842.4363 | Ib | OUTBREAK |  |
| SRR7163929 | RDNC |  |  |  |  | stx2c | 1.727.1041.2979.3665.3842.4363 | Ib | OUTBREAK |  |
| SRR7187892 | RDNC |  |  |  |  | stx2c | 1.727.1041.2979.3665.3842.4363 | Ib | OUTBREAK |  |
| SRR7209034 | RDNC |  |  |  |  | stx2c | 1.727.1041.2979.3665.3842.4363 | Ib | OUTBREAK |  |
| SRR7215572 | RDNC |  |  |  |  | stx2c | 1.727.1041.2979.3665.3842.4363 | Ib | OUTBREAK |  |
| SRR7265967 | RDNC |  |  |  |  | stx2c | 1.727.1041.2979.3665.3842.4363 | Ib | OUTBREAK |  |
| SRR7285472 | RDNC |  |  |  |  |  | 1.727.1041.2979.3665.3842.4363 | Ib | OUTBREAK |  |
| SRR6052786 | RDNC |  |  |  |  | stx2c | 1.727.1041.2979.3665.3842.4366 | Ib | OUTBREAK |  |
| SRR7291031 | RDNC |  |  |  |  | stx2c | 1.727.1041.2979.3665.3842.4366 | Ib | OUTBREAK |  |
| SRR7187908 | RDNC |  |  |  |  | stx2c | 1.727.1041.2979.3665.3842.4394 | Ib | OUTBREAK |  |
| SRR6052865 | RDNC |  |  |  |  | stx2c | 1.727.1041.2979.3665.3842.4621 | Ib | OUTBREAK |  |
| SRR7223036 | PT 31 | Yes | Yes | No | Yes | stx2c | 1.897.1658.3129.3919.4132.4743 | Ib |  |  |
| SRR7285519 | PT 31 | Yes | Yes | No | Yes | stx2c | 1.897.1658.3129.3919.4132.4743 | Ib |  |  |
| SRR6052871 | PT 8 | Yes | No | No | No | stx2c | 104.1078.1564.2993.3697.3876.4413 | IIa |  |  |
| SRR7230310 | PT 8 | Yes | No | No | No | stx2c | 104.1099.1657.3128.3918.4131.4742 | IIa |  |  |
| SRR7186892 | PT 1 | yes | No | Yes | No | stx2a stx1a | 11.147.1664.3144.3945.4165.4791 | Ia |  |  |
| SRR7167519 | PT 1 | yes | No | Yes | No | stx2a stx1a | 11.147.1664.3144.3945.4165.4805 | Ia |  |  |
| SRR7285437 | PT 23 | Yes | No | Yes | Yes | stx2a stx1a | 11.147.1714.3234.4082.4324.4995 | Ia |  |  |
| SRR6052082 | PT 14 | No | No | No | No | stx2a stx1a | 11.155.1523.2877.3536.3704.4203 | Ia |  |  |
| SRR6052996 | PT 32 | No | Yes | No | Yes | stx2a stx2c | 117.1095.1645.3109.3873.4083.4678 | Ib |  |  |
| SRR7184361 | PT 2 | No | No | No | Yes | stx2a stx2c | 117.1102.1665.3146.3948.4168.4794 | Ib |  |  |
| SRR7187916 | PT 2 | No | No | No | Yes | stx2a stx2c | 117.1102.1665.3146.3948.4203.4835 | Ib |  |  |
| SRR7291613 | PT 2 | No | No | No | Yes | stx2a stx2c | 117.1102.1665.3146.3948.4203.4835 | Ib |  |  |
| SRR6053036 | PT 14 | No | No | No | No | stx2a stx2c | 12.767.1100.2045.3719.3903.4454 | I/II |  |  |
| SRR6052122 | PT 8 | Yes | No | No | No | stx2c | 138.1055.1521.2873.3528.3696.4190 | IIa |  |  |
| SRR6053022 | PT 32 | No | Yes | No | Yes | stx2c | 139.1014.1435.3093.3847.4055.4643 | Ib |  |  |
| SRR6053035 | PT 32 | No | Yes | No | Yes | stx2c | 139.1014.1435.3093.3879.4090.4685 | Ib |  |  |
| SRR6052931 | PT 8 | Yes | No | No | No | stx2c | 14.1075.1557.2977.3663.3840.4359 | Outgroup |  |  |
| SRR6053034 | RDNC |  |  |  |  | stx2c | 14.1096.1646.3110.3874.4085.4680 | Outgroup |  |  |
| SRR7172299 | PT 8 | Yes | No | No | No | stx2c | 14.20.1308.2464.3907.4119.4721 | Outgroup |  |  |
| SRR5905747 | PT 34 | No | No | No | Yes | stx2c | 144.1038.1575.3013.3737.3925.4483 | IIa |  |  |
| SRR7187841 | PT 34 | No | No | No | Yes | stx2c | 144.1038.1575.3013.3737.3925.4483 | IIa |  |  |
| SRR6079429 | PT 34 | No | No | No | Yes | stx2c | 144.1038.1575.3060.3802.4002.4574 | IIa |  |  |
| SRR7265868 | PT 14 | No | No | No | No | stx2c stx1a | 15.22.1698.3200.4030.4265.4922 | IIb |  |  |
| SRR7283732 | PT 14 | No | No | No | No | stx2c | 159.1073.1551.2968.3644.3820.4335 | IIa |  |  |
| SRR7291626 | PT 21/28 | No | Yes | Yes | Yes | stx2c | 160.1076.1559.2981.3671.3848.4372 | IIa |  |  |
| SRR6052780 | PT 33 | Yes | Yes | Yes | Yes | stx2c | 160.1076.1559.2981.3671.3848.4404 | IIa |  |  |
| SRR7223033 | PT 14 | No | No | No | No | stx2c stx1a | 161.1082.1578.3019.3745.3935.4495 | IIb |  |  |
| SRR7265869 | PT 63 | Yes | No | Yes | Yes | stx2c | 163.1105.1679.3165.3981.4206.4839 | IIa |  |  |
| SRR7184236 | PT 2 | No | No | No | Yes | stx2c | 164.1106.1684.3177.3999.4226.4865 | IIa |  |  |
| SRR7283675 | PT 14 | No | No | No | No | stx2c stx1a | 165.1115.1707.3221.4064.4305.4973 | IIb |  |  |
| SRR6052789 | PT 14 | No | No | No | No | stx2c stx1a | 18.1063.1541.2956.3632.3807.4320 | IIb |  |  |
| SRR6816945 | PT 14 | No | No | No | No | stx2c stx1a | 18.1063.1703.3208.4042.4280.4940 | IIb |  |  |
| SRR6816940 | PT 4 | No | No | Yes | No | stx2c | 18.1097.1651.3116.3887.4098.4693 | IIb |  |  |
| SRR6816928 | PT 14 | No | No | No | No | stx2c | 18.35.1677.3163.3978.4201.4832 | IIb |  |  |
| SRR6816950 | PT 14 | No | No | No | No | stx2c | 18.35.1677.3163.3978.4201.4832 | IIb |  |  |
| SRR6816932 | PT 4 | No | No | Yes | No | stx2c | 18.35.1678.3164.3980.4204.4836 | IIb |  |  |
| SRR7401664 | PT 4 | No | No | Yes | No | stx2c | 18.35.1678.3164.3980.4204.4836 | IIb |  |  |
| SRR6816941 | PT 4 | No | No | Yes | No | stx2c | 18.35.275.2162.3417.4235.4880 | IIb |  |  |
| SRR6816931 | PT 4 | No | No | Yes | No | stx2c | 18.35.275.2162.3417.4235.4920 | IIb |  |  |
| SRR6816933 | PT 4 | No | No | Yes | No | stx2c | 18.35.275.3215.4055.4296.4962 | IIb |  |  |
| SRR6816929 | PT 8 | Yes | No | No | No | stx2a | 18.35.380.3131.3924.4139.4751 | IIb |  |  |
| SRR7215906 | PT 8 | Yes | No | No | No | stx2a | 18.35.380.3131.3924.4139.4751 | IIb |  |  |
| SRR7286548 | PT 8 | Yes | No | No | No | stx2a | 18.35.380.3131.3924.4139.4751 | IIb |  |  |
| SRR6816943 | PT 1 | yes | No | Yes | No |  | 18.35.380.3220.4062.4303.4971 | IIb |  |  |
| SRR6816946 | PT 1 | yes | No | Yes | No |  | 18.35.380.738.3915.4128.4738 | IIb |  |  |
| SRR6052705 | PT 8 | Yes | No | No | No | stx2a | 18.35.380.765.1009.1526.4352 | IIb | OUTBREAK |  |
| SRR6052743 | PT 8 | Yes | No | No | No | stx2a | 18.35.380.765.1009.1526.4352 | IIb | OUTBREAK |  |
| SRR6052863 | PT 8 | Yes | No | No | No | stx2a | 18.35.380.765.1009.1526.4626 | IIb | OUTBREAK |  |
| SRR6079442 | PT 8 | Yes | No | No | No | stx2a | 18.35.380.765.1009.1526.4647 | IIb | OUTBREAK |  |
| SRR6816939 | PT 8 | Yes | No | No | No | stx2a | 18.35.380.765.1009.1526.4960 | IIb | OUTBREAK |  |
| SRR6816954 | PT 8 | Yes | No | No | No | stx2a | 18.35.380.765.1009.1526.5002 | IIb | OUTBREAK |  |
| SRR7285564 | PT 8 | Yes | No | No | No | stx2a | 18.35.380.765.1009.1526.5002 | IIb | OUTBREAK |  |
| SRR7286636 | PT 8 | Yes | No | No | No | stx2a | 18.35.380.765.1009.1526.5002 | IIb | OUTBREAK |  |
| SRR6079424 | PT 8 | Yes | No | No | No | stx2a | 18.35.380.765.1009.3264.4510 | IIb |  |  |
| SRR6053044 | PT 8 | Yes | No | No | No | stx2a | 18.35.380.765.1009.3345.4584 | IIb |  |  |
| SRR6821014 | PT 8 | Yes | No | No | No | stx2a | 18.35.380.765.1009.3717.4216 | IIb |  |  |
| SRR6052890 | PT 8 | Yes | No | No | No | stx2a | 18.35.380.765.1009.4023.4600 | IIb |  |  |
| SRR6825176 | PT 8 | Yes | No | No | No | stx2a | 18.35.380.765.2572.4008.4581 | IIb |  |  |
| SRR6053028 | PT 8 | Yes | No | No | No | stx2a | 18.35.380.765.2572.4008.4622 | IIb |  |  |
| SRR6821015 | PT 67 | Yes | No | Yes | Yes | stx2a | 18.35.380.765.2572.4016.4593 | IIb |  |  |
| SRR6052917 | PT 23 | Yes | No | Yes | Yes | stx2a | 18.35.380.765.2572.4016.4601 | IIb |  |  |
| SRR6052924 | PT 8 | Yes | No | No | No | stx2a | 18.35.380.765.2572.4084.4679 | IIb |  |  |
| SRR6052126 | PT 1 | yes | No | Yes | No | stx2a | 18.35.380.765.3534.3702.4197 | IIb |  |  |
| SRR6052739 | PT 1 | yes | No | Yes | No | stx2a | 18.35.380.765.3650.3826.4342 | IIb |  |  |
| SRR6052773 | PT 1 | yes | No | Yes | No | stx2a | 18.35.380.765.3650.3826.4342 | IIb |  |  |
| SRR6052910 | PT 1 | yes | No | Yes | No | stx2a | 18.35.380.765.3650.4056.4644 | IIb |  |  |
| SRR6816937 | PT 1 | yes | No | Yes | No | stx2a | 18.35.380.765.3650.4056.4644 | IIb |  |  |
| SRR6820993 | PT 1 | yes | No | Yes | No | stx2a | 18.35.380.765.3650.4056.4644 | IIb |  |  |
| SRR7204324 | PT 1 | yes | No | Yes | No | stx2a | 18.35.380.765.3650.4056.4644 | IIb |  |  |
| SRR6825174 | PT 1 | yes | No | Yes | No | stx2a | 18.35.380.765.3650.4056.4669 | IIb |  |  |
| SRR6816949 | PT 1 | yes | No | Yes | No | stx2a | 18.35.380.765.3650.4056.4757 | IIb |  |  |
| SRR6052862 | PT 1 | yes | No | Yes | No | stx2a | 18.35.380.765.3660.3837.4481 | IIb |  |  |
| SRR6053018 | PT 8 | Yes | No | No | No | stx2a | 18.35.380.765.3675.3853.4377 | IIb |  |  |
| SRR6052907 | PT 1 | yes | No | Yes | No | stx2a | 18.35.380.765.3706.3887.4430 | IIb |  |  |
| SRR6052926 | PT 1 | yes | No | Yes | No | stx2a | 18.35.380.765.3804.4006.4579 | IIb |  |  |
| SRR6052864 | PT 1 | yes | No | Yes | No | stx2a | 18.35.380.765.3826.4033.4611 | IIb |  |  |
| SRR6825177 | PT 1 | yes | No | Yes | No | stx2a | 18.35.380.765.3826.4033.4611 | IIb |  |  |
| SRR6052928 | PT 1 | yes | No | Yes | No | stx2a | 18.35.380.765.3833.4041.4624 | IIb |  |  |
| SRR6816938 | PT 8 | Yes | No | No | No | stx2a | 18.35.380.765.3880.4091.4686 | IIb |  |  |
| SRR6816935 | PT 8 | Yes | No | No | No | stx2a | 18.35.380.765.3914.4127.4737 | IIb |  |  |
| SRR6816930 | PT 8 | Yes | No | No | No | stx2a | 18.35.380.765.3951.4171.4797 | IIb |  |  |
| SRR6816944 | PT 1 | yes | No | Yes | No | stx2a | 18.35.380.765.3968.4190.4819 | IIb |  |  |
| SRR6816936 | PT 8 | Yes | No | No | No | stx2a | 18.35.380.765.3989.4214.4853 | IIb |  |  |
| SRR7277736 | PT 8 | Yes | No | No | No | stx2a | 18.35.380.765.3989.4214.4853 | IIb |  |  |
| SRR7407974 | PT 8 | Yes | No | No | No | stx2a | 18.35.380.765.3989.4214.4853 | IIb |  |  |
| SRR7236579 | PT 8 | Yes | No | No | No | stx2a | 18.35.380.765.3989.4214.4886 | IIb |  |  |
| SRR6816955 | PT 1 | yes | No | Yes | No | stx2a | 18.35.380.765.3994.4220.4859 | IIb |  |  |
| SRR7290966 | PT 1 | yes | No | Yes | No | stx2a | 18.35.380.765.3997.4224.4863 | IIb |  |  |
| SRR7290926 | PT 14 | No | No | No | No | stx2a | 18.35.380.765.4015.4246.4897 | IIb |  |  |
| SRR6821009 | PT 4 | No | No | Yes | No | stx2c | 18.35.445.3011.3731.3917.4471 | IIb |  |  |
| SRR6820839 | PT 4 | No | No | Yes | No | stx2c | 18.35.445.842.3754.3945.4507 | IIb |  |  |
| SRR6052879 | PT 4 | No | No | Yes | No | stx2c | 18.35.713.1310.3693.3872.4408 | IIb |  |  |
| SRR6821012 | PT 4 | No | No | Yes | No | stx2c | 18.35.972.1802.3761.3956.4520 | IIb |  |  |
| SRR6052857 | PT 4 | No | No | Yes | No | stx2c | 18.35.972.1802.3830.4037.4616 | IIb |  |  |
| SRR6052893 | PT 4 | No | No | Yes | No | stx2c | 18.35.972.1802.3830.4037.4616 | IIb |  |  |
| SRR6825173 | PT 4 | No | No | Yes | No | stx2c | 18.35.972.1802.3830.4037.4616 | IIb |  |  |
| SRR6825175 | PT 4 | No | No | Yes | No | stx2c | 18.35.972.1802.3830.4037.4616 | IIb |  |  |
| SRR6816951 | PT 2 | No | No | No | Yes | stx2a stx2c | 18.444.1673.3156.3967.4189.4818 | IIb |  |  |
| SRR6052719 | PT 2 | No | No | No | Yes | stx2a stx2c | 18.444.673.1244.3540.3708.4207 | IIb |  |  |
| SRR6816934 | PT 2 | No | No | No | Yes | stx2a stx2c | 18.444.673.1244.3540.3708.4207 | IIb |  |  |
| SRR6816956 | PT 2 | No | No | No | Yes | stx2a stx2c | 18.444.673.1244.3540.3708.4207 | IIb |  |  |
| SRR6816942 | PT 2 | No | No | No | Yes | stx2a stx2c | 18.444.673.1244.3903.4115.4717 | IIb |  |  |
| SRR7186948 | PT 2 | No | No | No | Yes | stx2a stx2c | 18.444.673.1244.3903.4115.4717 | IIb |  |  |
| SRR7283770 | PT 2 | No | No | No | Yes | stx2a stx2c | 18.444.673.1244.3903.4115.4717 | IIb |  |  |
| SRR7274859 | PT 8 | Yes | No | No | No | stx2c stx1a | 2.1091.1607.3055.3796.3995.4565 | IIc |  |  |
| SRR6052830 |  |  |  |  |  | stx2a stx2c stx1a | 2.154.1568.3000.3709.3891.4441 | IIc |  |  |
| SRR6052844 | PT 8 | Yes | No | No | No | stx2c stx1a | 2.154.1572.3009.3728.3914.4466 | IIc |  |  |
| SRR7172298 | PT 8 | Yes | No | No | No | stx2c stx1a | 2.154.1610.3065.3810.4012.4587 | IIc |  |  |
| SRR7251474 | PT 8 | Yes | No | No | No | stx2c stx1a | 2.154.1661.3140.3938.4156.4777 | IIc |  |  |
| SRR7251488 | PT 8 | Yes | No | No | No | stx2c stx1a | 2.154.1661.3140.3938.4156.4777 | IIc |  |  |
| SRR7230302 | PT 8 | Yes | No | No | No | stx2c stx1a | 2.154.306.1963.4057.4298.4964 | IIc |  |  |
| SRR7276946 | PT 8 | Yes | No | No | No | stx2c stx1a | 2.154.306.1963.4057.4298.4964 | IIc |  |  |
| SRR7285540 | PT 8 | Yes | No | No | No | stx2c stx1a | 2.154.306.1963.4057.4298.4964 | IIc |  |  |
| SRR7286582 | PT 8 | Yes | No | No | No | stx2c stx1a | 2.154.306.1963.4057.4298.4964 | IIc |  |  |
| SRR7367379 | PT 8 | Yes | No | No | No | stx2c stx1a | 2.154.306.1963.4057.4298.4964 | IIc |  |  |
| SRR7368879 | PT 8 | Yes | No | No | No | stx2c stx1a | 2.154.306.1963.4057.4298.4964 | IIc |  |  |
| SRR7184350 | PT 8 | Yes | No | No | No | stx2c stx1a | 2.154.306.1963.4057.4298.4986 | IIc |  |  |
| SRR7163886 | PT 8 | Yes | No | No | No | stx2c stx1a | 2.154.306.2082.3979.4202.4833 | IIc |  |  |
| SRR6052794 | PT 8 | Yes | No | No | No | stx2c stx1a | 2.154.306.2140.3750.3941.4501 | IIc |  |  |
| SRR7265979 | PT 8 | Yes | No | No | No | stx2c stx1a | 2.154.306.2140.3750.3941.4501 | IIc |  |  |
| SRR6052776 | PT 8 | Yes | No | No | No | stx2c stx1a | 2.154.306.2983.3677.3855.4381 | IIc |  |  |
| SRR6052770 | PT 8 | Yes | No | No | No | stx2c stx1a | 2.154.306.2983.3677.3855.4382 | IIc |  |  |
| SRR6052775 | PT 8 | Yes | No | No | No | stx2c stx1a | 2.154.306.2990.3688.3867.4401 | IIc |  |  |
| SRR6001305 | PT 8 | Yes | No | No | No | stx2c stx1a | 2.154.306.3003.3715.3899.4450 | IIc |  |  |
| SRR6053039 | PT 8 | Yes | No | No | No | stx2c stx1a | 2.154.306.3003.3715.3899.4450 | IIc |  |  |
| SRR7285508 | PT 8 | Yes | No | No | No | stx2c stx1a | 2.154.306.3003.3715.3899.4450 | IIc |  |  |
| SRR6052995 | PT 8 | Yes | No | No | No | stx2c stx1a | 2.154.306.3007.3724.3909.4460 | IIc |  |  |
| SRR6053030 | PT 8 | Yes | No | No | No | stx2c stx1a | 2.154.306.3007.3724.3909.4473 | IIc |  |  |
| SRR6052870 | PT 8 | Yes | No | No | No | stx2c stx1a | 2.154.306.3094.3850.4058.4648 | IIc |  |  |
| SRR7167525 | PT 8 | Yes | No | No | No | stx2c stx1a | 2.154.306.3122.3906.4118.4720 | IIc |  |  |
| SRR7223135 | PT 8 | Yes | No | No | No | stx2c stx1a | 2.154.306.3122.3906.4118.4724 | IIc |  |  |
| SRR7286379 | PT 8 | Yes | No | No | No | stx2c stx1a | 2.154.306.3122.3906.4118.4729 | IIc |  |  |
| SRR7191575 | PT 8 | Yes | No | No | No | stx2c stx1a | 2.154.306.3122.3906.4118.4739 | IIc |  |  |
| SRR7286628 | PT 8 | Yes | No | No | No | stx2c stx1a | 2.154.306.3139.3934.4150.4768 | IIc |  |  |
| SRR7163885 | PT 8 | Yes | No | No | No | stx2c stx1a | 2.154.306.3162.3977.4200.4831 | IIc |  |  |
| SRR7251463 | PT 8 | Yes | No | No | No | stx2c stx1a | 2.154.306.3230.4075.4317.4987 | IIc |  |  |
| SRR6052754 | PT 8 | Yes | No | No | No | stx2c stx1a | 2.154.306.629.3648.3824.4396 | IIc |  |  |
| SRR6052887 | PT 8 | Yes | No | No | No | stx2c stx1a | 2.154.306.629.3757.3951.4515 | IIc |  |  |
| SRR7285703 | PT 8 | Yes | No | No | No | stx2c stx1a | 2.154.315.2041.3812.4014.4590 | IIc |  |  |
| SRR6001300 | PT 8 | Yes | No | No | No | stx2c stx1a | 2.8.1078.2472.3718.3902.4453 | IIc |  |  |
| SRR6001336 | PT 8 | Yes | No | No | No | stx2c stx1a | 2.8.1108.2064.2403.3130.4386 | IIc |  |  |
| SRR6052779 | PT 8 | Yes | No | No | No | stx2c stx1a | 2.8.1108.2064.2403.3130.4386 | IIc |  |  |
| SRR6053032 | PT 8 | Yes | No | No | No | stx2c stx1a | 2.8.1108.2064.3808.4010.4585 | IIc |  |  |
| SRR6053038 | PT 8 | Yes | No | No | No | stx2c stx1a | 2.8.1108.2655.3169.4040.4623 | IIc |  |  |
| SRR7184327 | PT 54 | Yes | No | No | Yes | stx2c stx1a | 2.8.1108.2655.3169.4163.4789 | IIc |  |  |
| SRR6052821 | PT 8 | Yes | No | No | No | stx2a stx1a | 2.8.1112.2068.3852.4060.4650 | IIc |  |  |
| SRR6052847 | PT 8 | Yes | No | No | No | stx2a stx1a | 2.8.1112.2068.3852.4060.4650 | IIc |  |  |
| SRR6079428 | PT 14 | No | No | No | No | stx2c stx1a | 2.8.1172.2169.3712.3894.4445 | IIc |  |  |
| SRR7283816 | PT 14 | No | No | No | No | stx2c stx1a | 2.8.1172.2169.3712.3894.4445 | IIc |  |  |
| SRR7230313 | PT 8 | Yes | No | No | No | stx2c stx1a | 2.8.1240.2290.3952.4172.4798 | IIc |  |  |
| SRR7286173 | PT 8 | Yes | No | No | No | stx2c stx1a | 2.8.1250.3022.3748.3939.4499 | IIc |  |  |
| SRR6079447 | PT 8 | Yes | No | No | No | stx2c stx1a | 2.8.1291.3043.3779.3977.4543 | IIc |  |  |
| SRR6079364 | PT 8 | Yes | No | No | No | stx2a stx2c stx1a | 2.8.1343.2869.3524.3691.4185 | IIc |  |  |
| SRR6052914 | PT 8 | Yes | No | No | No | stx2c stx1a | 2.8.1343.3082.3832.4039.4620 | IIc |  |  |
| SRR7401659 | PT 14 | No | No | No | No | stx2c stx1a | 2.8.1358.2548.3044.3165.4884 | IIc |  |  |
| SRR6052869 | PT 8 | Yes | No | No | No | stx2c stx1a | 2.8.1565.2994.3698.3877.4419 | IIc |  |  |
| SRR7186939 | PT 8 | Yes | No | No | No | stx2c stx1a | 2.8.1565.2994.3698.3877.4422 | IIc |  |  |
| SRR6001323 | PT 8 | Yes | No | No | No | stx2c stx1a | 2.8.1567.2997.3704.3885.4428 | IIc |  |  |
| SRR7251007 | PT 8 | Yes | No | No | No | stx2c stx1a | 2.8.1567.2997.3704.3885.4428 | IIc |  |  |
| SRR6053017 | PT 8 | Yes | No | No | No | stx2c stx1a | 2.8.1567.2997.3704.3885.4436 | IIc |  |  |
| SRR6053010 | PT 8 | Yes | No | No | No | stx2c stx1a | 2.8.1576.3015.3740.3929.4488 | IIc |  |  |
| SRR6053031 | PT 8 | Yes | No | No | No | stx2c stx1a | 2.8.1576.3015.3740.3929.4506 | IIc |  |  |
| SRR6079446 | PT 8 | Yes | No | No | No | stx2c stx1a | 2.8.1624.3069.3818.4022.4599 | IIc |  |  |
| SRR6052905 | PT 8 | Yes | No | No | No | stx2c stx1a | 2.8.1629.3077.3827.4034.4613 | IIc |  |  |
| SRR7290992 | PT 8 | Yes | No | No | No | stx2c stx1a | 2.8.1629.3077.3827.4034.4618 | IIc |  |  |
| SRR7187765 | PT 8 | Yes | No | No | No | stx2c stx1a | 2.8.1631.3081.3831.4038.4619 | IIc |  |  |
| SRR6052895 | PT 8 | Yes | No | No | No | stx2c stx1a | 2.8.1633.3084.3835.4043.4627 | IIc |  |  |
| SRR6052853 | PT 8 | Yes | No | No | No | stx2c stx1a | 2.8.1637.3095.3851.4059.4649 | IIc |  |  |
| SRR7286598 | RDNC |  |  |  |  | stx2c stx1a | 2.8.1639.3097.3855.4064.4654 | IIc |  |  |
| SRR7179947 | PT 8 | Yes | No | No | No | stx2c stx1a | 2.8.1643.3102.3863.4072.4666 | IIc |  |  |
| SRR6052823 | PT 8 | Yes | No | No | No | stx2c stx1a | 2.8.1648.3112.3878.4089.4684 | IIc |  |  |
| SRR7223161 | PT 8 | Yes | No | No | No | stx2c stx1a | 2.8.1650.3115.3886.4097.4692 | IIc |  |  |
| SRR7204298 | PT 8 | Yes | No | No | No | stx2c stx1a | 2.8.1650.3115.4016.4247.4898 | IIc |  |  |
| SRR7286630 | PT 8 | Yes | No | No | No | stx2c stx1a | 2.8.1652.3118.3889.4101.4697 | IIc |  |  |
| SRR7286588 | PT 14 | No | No | No | No | stx2c stx1a | 2.8.1653.3119.3890.4102.4698 | IIc |  |  |
| SRR7204337 | PT 8 | Yes | No | No | No | stx2c stx1a | 2.8.1708.3222.4066.4307.4975 | IIc |  |  |
| SRR7283671 | PT 8 | Yes | No | No | No | stx2a stx2c stx1a | 2.8.25.1027.3649.3825.4341 | IIc |  |  |
| SRR6079386 | PT 8 | Yes | No | No | No | stx2a stx2c stx1a | 2.8.25.1027.3649.3825.4345 | IIc |  |  |
| SRR7249679 | PT 8 | Yes | No | No | No | stx2c stx1a | 2.8.25.1933.4113.4356.5033 | IIc |  |  |
| SRR7285444 | PT 8 | Yes | No | No | No | stx2c stx1a | 2.8.25.2291.2667.2759.3350 | IIc |  |  |
| SRR7401632 | PT 8 | Yes | No | No | No | stx2c stx1a | 2.8.25.2291.2667.2759.4199 | IIc |  |  |
| SRR7286400 | PT 8 | Yes | No | No | No | stx2c stx1a | 2.8.25.2419.3027.3145.4728 | IIc |  |  |
| SRR7282609 | PT 8 | Yes | No | No | No | stx2c stx1a | 2.8.25.2419.3027.3145.4834 | IIc |  |  |
| SRR6052878 | PT 8 | Yes | No | No | No | stx1a | 2.8.25.2419.3875.4086.4681 | IIc |  |  |
| SRR6079441 | PT 8 | Yes | No | No | No | stx2c stx1a | 2.8.25.2490.2952.3068.4612 | IIc |  |  |
| SRR7244294 | PT 8 | Yes | No | No | No | stx1a | 2.8.25.2514.4108.4351.5028 | IIc |  |  |
| SRR6079415 | PT 8 | Yes | No | No | No | stx2c stx1a | 2.8.25.2987.3684.3862.4395 | IIc |  |  |
| SRR7215519 | PT 8 | Yes | No | No | No | stx2c stx1a | 2.8.25.3017.3743.3932.4491 | IIc |  |  |
| SRR6079433 | PT 8 | Yes | No | No | No | stx2c stx1a | 2.8.25.3017.3743.3932.4557 | IIc |  |  |
| SRR7223127 | PT 8 | Yes | No | No | No | stx2c stx1a | 2.8.25.3017.3743.3932.4838 | IIc |  |  |
| SRR6052913 | PT 8 | Yes | No | No | No | stx2c stx1a | 2.8.25.3018.3744.3934.4493 | IIc |  |  |
| SRR7250977 | PT 8 | Yes | No | No | No | stx2c stx1a | 2.8.25.3018.3744.3934.4493 | IIc |  |  |
| SRR7244239 | PT 8 | Yes | No | No | No | stx2c stx1a | 2.8.25.625.3949.4169.4795 | IIc |  |  |
| SRR7401634 | PT 8 | Yes | No | No | No | stx2c stx1a | 2.8.324.644.888.2642.4417 | IIc |  |  |
| SRR7184207 | PT 8 | Yes | No | No | No | stx2c stx1a | 2.8.324.644.888.2642.4903 | IIc |  |  |
| SRR7184382 | PT 8 | Yes | No | No | No | stx2c stx1a | 2.8.324.644.888.2642.4903 | IIc |  |  |
| SRR7186899 | PT 8 | Yes | No | No | No | stx2c stx1a | 2.8.324.644.888.2642.4903 | IIc |  |  |
| SRR7187889 | PT 8 | Yes | No | No | No | stx2c stx1a | 2.8.324.644.888.2642.4903 | IIc |  |  |
| SRR7236626 | PT 8 | Yes | No | No | No | stx2c stx1a | 2.8.324.644.888.2642.4903 | IIc |  |  |
| SRR7215278 | PT 8 | Yes | No | No | No | stx2c stx1a | 2.8.324.644.888.2642.4952 | IIc |  |  |
| SRR7290990 | PT 8 | Yes | No | No | No | stx2c stx1a | 2.8.327.1718.3807.4009.4582 | IIc |  |  |
| SRR6052840 | PT 8 | Yes | No | No | No | stx2c stx1a | 2.8.327.1718.3859.4068.4660 | IIc |  |  |
| SRR7251452 | PT 8 | Yes | No | No | No | stx2c stx1a | 2.8.327.1718.3859.4068.4660 | IIc |  |  |
| SRR7274961 | PT 8 | Yes | No | No | No | stx2c stx1a | 2.8.327.1718.3859.4068.4660 | IIc |  |  |
| SRR7184208 | PT 8 | Yes | No | No | No | stx2c stx1a | 2.8.327.1718.3947.4167.4793 | IIc |  |  |
| SRR7221250 | PT 8 | Yes | No | No | No | stx2c stx1a | 2.8.327.1718.4061.4302.4970 | IIc |  |  |
| SRR6079431 | PT 8 | Yes | No | No | No | stx2c stx1a | 2.8.327.2077.3230.3378.3808 | IIc |  |  |
| SRR6052906 | PT 8 | Yes | No | No | No | stx2c stx1a | 2.8.327.2189.3742.3931.4490 | IIc |  |  |
| SRR7291673 | PT 8 | Yes | No | No | No | stx2c stx1a | 2.8.327.2227.3894.4106.4707 | IIc |  |  |
| SRR6052764 | PT 8 | Yes | No | No | No | stx2c stx1a | 2.8.327.2258.3554.3723.4224 | IIc |  |  |
| SRR7265839 | PT 8 | Yes | No | No | No | stx2c stx1a | 2.8.327.2258.3554.3723.4224 | IIc |  |  |
| SRR6053007 | PT 8 | Yes | No | No | No | stx2c stx1a | 2.8.327.2685.3683.3861.4392 | IIc |  |  |
| SRR6052807 | PT 8 | Yes | No | No | No | stx2c stx1a | 2.8.327.2986.3681.3859.4389 | IIc |  |  |
| SRR6052781 | PT 8 | Yes | No | No | No | stx2c stx1a | 2.8.327.2992.3694.3873.4410 | IIc |  |  |
| SRR6052909 | PT 8 | Yes | No | No | No | stx2c stx1a | 2.8.327.3014.3739.3927.4486 | IIc |  |  |
| SRR7191463 | PT 8 | Yes | No | No | No | stx2c stx1a | 2.8.327.3014.3739.3927.4486 | IIc |  |  |
| SRR6053019 | PT 8 | Yes | No | No | No | stx2a stx2c stx1a | 2.8.327.3106.3869.4079.4674 | IIc |  |  |
| SRR7187839 | PT 8 | Yes | No | No | No | stx2c stx1a | 2.8.327.3154.3964.4186.4815 | IIc |  |  |
| SRR7291636 | PT 8 | Yes | No | No | No | stx2c stx1a | 2.8.327.3181.4007.4237.4882 | IIc |  |  |
| SRR7286253 | PT 8 | Yes | No | No | No | stx2c stx1a | 2.8.327.824.3306.3461.4640 | IIc |  |  |
| SRR6052849 | PT 8 | Yes | No | No | No | stx2c stx1a | 2.8.327.824.3795.3994.4562 | IIc |  |  |
| SRR7358276 | PT 8 | Yes | No | No | No | stx2c stx1a | 2.8.327.824.3943.4162.4788 | IIc |  |  |
| SRR7186954 | PT 8 | Yes | No | No | No | stx2c stx1a | 2.8.327.824.3943.4162.4807 | IIc |  |  |
| SRR7187004 | PT 8 | Yes | No | No | No | stx2c stx1a | 2.8.327.824.3943.4162.4841 | IIc |  |  |
| SRR7283821 | PT 8 | Yes | No | No | No | stx2c stx1a | 2.8.334.3202.4033.4269.4927 | IIc |  |  |
| SRR7221343 | PT 8 | Yes | No | No | No | stx2c stx1a | 2.8.334.661.3247.4099.4694 | IIc |  |  |
| SRR7266826 | PT 8 | Yes | No | No | No | stx2c stx1a | 2.8.334.661.3247.4099.4694 | IIc |  |  |
| SRR7367576 | PT 8 | Yes | No | No | No | stx2c stx1a | 2.8.334.661.3247.4099.4694 | IIc |  |  |
| SRR7163915 | PT 31 | Yes | Yes | No | Yes | stx2c stx1a | 2.8.334.661.3247.4099.4694 | IIc |  |  |
| SRR7367550 | PT 8 | Yes | No | No | No | stx2c stx1a | 2.8.334.661.3247.4099.4845 | IIc |  |  |
| SRR7167526 | PT 31 | Yes | Yes | No | Yes | stx2c stx1a | 2.8.334.661.3247.4099.5003 | IIc |  |  |
| SRR6052736 | PT 8 | Yes | No | No | No | stx2c stx1a | 2.8.334.661.3647.3823.4338 | IIc |  |  |
| SRR6052124 | PT 54 | Yes | No | No | Yes | stx2c stx1a | 2.8.343.1732.2180.3681.4167 | IIc |  |  |
| SRR6052911 | PT 54 | Yes | No | No | Yes | stx2c stx1a | 2.8.343.1732.2180.3681.4448 | IIc |  |  |
| SRR7236633 | PT 54 | Yes | No | No | Yes | stx2c stx1a | 2.8.343.1732.2180.3681.5025 | IIc |  |  |
| SRR7291584 | PT 54 | Yes | No | No | Yes | stx2c stx1a | 2.8.343.1732.2180.3681.5025 | IIc |  |  |
| SRR6052814 | PT 54 | Yes | No | No | Yes | stx2c stx1a | 2.8.343.1732.3721.3905.4456 | IIc |  |  |
| SRR6052826 | PT 54 | Yes | No | No | Yes | stx2c stx1a | 2.8.343.1732.3721.3905.4456 | IIc |  |  |
| SRR6052835 | PT 54 | Yes | No | No | Yes | stx2c stx1a | 2.8.343.1732.3721.3905.4456 | IIc |  |  |
| SRR6052860 | PT 54 | Yes | No | No | Yes | stx2c stx1a | 2.8.343.1732.3721.3905.4456 | IIc |  |  |
| SRR6053000 | PT 54 | Yes | No | No | Yes | stx2c stx1a | 2.8.343.1732.3721.3905.4456 | IIc |  |  |
| SRR6079419 | PT 54 | Yes | No | No | Yes | stx2c stx1a | 2.8.343.1732.3721.3905.4456 | IIc |  |  |
| SRR6052833 | PT 54 | Yes | No | No | Yes | stx2c stx1a | 2.8.343.1732.3721.3905.4485 | IIc |  |  |
| SRR7285524 | PT 54 | Yes | No | No | Yes | stx2c stx1a | 2.8.343.1732.3900.4112.4714 | IIc |  |  |
| SRR7221192 | PT 54 | Yes | No | No | Yes | stx2c stx1a | 2.8.343.1732.3900.4255.4910 | IIc |  |  |
| SRR7265740 | PT 54 | Yes | No | No | Yes | stx2c stx1a | 2.8.343.1732.3900.4255.4937 | IIc |  |  |
| SRR7285451 | PT 54 | Yes | No | No | Yes | stx2c stx1a | 2.8.343.1732.3990.4215.4854 | IIc |  |  |
| SRR7367541 | PT 54 | Yes | No | No | Yes | stx2c stx1a | 2.8.343.1732.3990.4215.4854 | IIc |  |  |
| SRR7285509 | PT 54 | Yes | No | No | Yes | stx2c stx1a | 2.8.343.1732.3990.4215.4923 | IIc |  |  |
| SRR7286255 | PT 54 | Yes | No | No | Yes | stx2c stx1a | 2.8.343.1732.4090.4333.5007 | IIc |  |  |
| SRR6052741 | PT 54 | Yes | No | No | Yes | stx2c stx1a | 2.8.343.2397.2820.2928.3216 | IIc |  |  |
| SRR6052735 | PT 8 | Yes | No | No | No | stx2c stx1a | 2.8.343.2397.2820.2928.4312 | IIc |  |  |
| SRR7277698 | PT 54 | Yes | No | No | Yes | stx2c stx1a | 2.8.343.2397.2820.2928.4334 | IIc |  |  |
| SRR6052802 | RDNC |  |  |  |  | stx2c stx1a | 2.8.343.2397.2820.2928.4416 | IIc |  |  |
| SRR6052756 | RDNC |  |  |  |  | stx2c | 2.8.343.2768.3382.3524.3965 | IIc |  |  |
| SRR7187790 | PT 8 | Yes | No | No | No | stx2a stx2c stx1a | 2.8.350.3117.3888.4100.4695 | IIc |  |  |
| SRR7283798 | PT 8 | Yes | No | No | No | stx2c stx1a | 2.8.351.2239.3986.4211.4847 | IIc |  |  |
| SRR7191711 | PT 8 | Yes | No | No | No | stx2c stx1a | 2.8.351.3213.4051.4291.4955 | IIc |  |  |
| SRR6052710 | PT 1 | yes | No | Yes | No | stx2c stx1a | 2.8.351.691.2957.3073.3389 | IIc |  |  |
| SRR6052718 | PT 1 | yes | No | Yes | No | stx2c stx1a | 2.8.351.691.2957.3073.3389 | IIc |  |  |
| SRR6052778 | PT 8 | Yes | No | No | No | stx2c stx1a | 2.8.351.691.3672.3849.4373 | IIc |  |  |
| SRR7367469 | PT 8 | Yes | No | No | No | stx2c stx1a | 2.8.351.691.3897.4109.4711 | IIc |  |  |
| SRR7251048 | PT 8 | Yes | No | No | No | stx2c stx1a | 2.8.351.691.3897.4109.4746 | IIc |  |  |
| SRR7251437 | PT 8 | Yes | No | No | No | stx2c stx1a | 2.8.351.691.4065.4306.4974 | IIc |  |  |
| SRR7221193 | PT 8 | Yes | No | No | No | stx2c stx1a | 2.8.351.812.2518.4178.4804 | IIc |  |  |
| SRR7367547 | PT 8 | Yes | No | No | No | stx2c stx1a | 2.8.351.812.3960.4181.4809 | IIc |  |  |
| SRR6053011 | PT 8 | Yes | No | No | No | stx2c stx1a | 2.8.393.3020.3746.3937.4497 | IIc |  |  |
| SRR7187875 | PT 54 | Yes | No | No | Yes | stx2c stx1a | 2.8.413.2272.2640.2730.4081 | IIc |  |  |
| SRR7187801 | PT 54 | Yes | No | No | Yes | stx1a | 2.8.413.2272.2640.2730.4222 | IIc |  |  |
| SRR6052102 | PT 54 | Yes | No | No | Yes | stx2c stx1a | 2.8.413.2272.2640.3249.4171 | IIc |  |  |
| SRR7184359 | PT 54 | Yes | No | No | Yes | stx2c stx1a | 2.8.413.2272.2640.3249.4171 | IIc |  |  |
| SRR6052121 | PT 54 | Yes | No | No | Yes | stx2c stx1a | 2.8.413.2272.2640.3249.4226 | IIc |  |  |
| SRR6079365 | PT 54 | Yes | No | No | Yes | stx2c stx1a | 2.8.413.2272.2640.3756.4260 | IIc |  |  |
| SRR7223137 | PT 8 | Yes | No | No | No | stx2c stx1a | 2.8.561.2074.2377.4218.4860 | IIc |  |  |
| SRR7208791 | PT 8 | Yes | No | No | No | stx2c stx1a | 2.8.960.2076.3815.4018.4595 | IIc |  |  |
| SRR7401642 | PT 8 | Yes | No | No | No | stx2c stx1a | 2.8.960.2076.3815.4018.4617 | IIc |  |  |
| SRR6052832 | PT 8 | Yes | No | No | No | stx2c stx1a | 2.8.960.3111.3877.4088.4683 | IIc |  |  |
| SRR7215509 | PT 8 | Yes | No | No | No | stx2c stx1a | 2.8.960.3121.3904.4116.4718 | IIc |  |  |
| SRR7283749 | PT 8 | Yes | No | No | No | stx2c stx1a | 2.807.1316.3192.4022.4253.4908 | IIc |  |  |
| SRR7285482 | PT 8 | Yes | No | No | No | stx2c stx1a | 2.807.1644.3103.3864.4073.4667 | IIc |  |  |
| SRR7285523 | PT 8 | Yes | No | No | No | stx2c stx1a | 2.807.1644.3103.3864.4073.4667 | IIc |  |  |
| SRR7286696 | PT 8 | Yes | No | No | No | stx2c stx1a | 2.807.1644.3103.3864.4073.4667 | IIc |  |  |
| SRR7286521 | PT 8 | Yes | No | No | No | stx2c stx1a | 2.807.1680.3166.3982.4207.4840 | IIc |  |  |
| SRR7285551 | PT 10 | No | No | Yes | No | stx2a stx2c stx1a | 24.769.1102.3188.4018.4249.4902 | IIb |  |  |
| SRR6001314 | PT 8 | Yes | No | No | No | stx2c | 3.735.1538.2952.3627.3801.4309 | IIa |  |  |
| SRR7204322 | PT 14 | No | No | No | No | stx2c | 3.735.1538.2952.3627.3801.4309 | IIa |  |  |
| SRR6053008 | PT 14 | No | No | No | No | stx2c | 3.87.1491.3068.3817.4021.4598 | IIa |  |  |
| SRR6052843 | PT 54 | Yes | No | No | Yes | stx2c | 39.1083.1579.3021.3747.3938.4498 | IIa |  |  |
| SRR7277790 | PT 32 | No | Yes | No | Yes | stx2a stx2c | 4.1100.1659.3132.3925.4141.4753 | Ic |  |  |
| SRR7172290 | PT 21/28 | No | Yes | Yes | Yes | stx2a stx2c | 4.4.1165.3182.4008.4238.4883 | Ic |  |  |
| SRR6052793 | PT 21/28 | No | Yes | Yes | Yes | stx2a | 4.4.1569.3001.3710.3892.4442 | Ic |  |  |
| SRR7367468 | PT 21/28 | No | Yes | Yes | Yes | stx2a | 4.4.1569.3001.3710.3892.4782 | Ic |  |  |
| SRR7265941 | PT 21/28 | No | Yes | Yes | Yes | stx2a | 4.4.1569.3253.4112.4355.5032 | Ic |  |  |
| SRR7282603 | PT 21/28 | No | Yes | Yes | Yes | stx2a stx2c | 4.4.1609.3063.3805.4007.4580 | Ic |  |  |
| SRR7256983 | PT 1 | yes | No | Yes | No | stx2a stx2c | 4.4.1641.3100.3861.4070.4663 | Ic |  |  |
| SRR7256994 | PT 1 | yes | No | Yes | No | stx2a stx2c | 4.4.1641.3100.3861.4070.4665 | Ic |  |  |
| SRR7244288 | PT 1 | yes | No | Yes | No | stx2a stx2c | 4.4.1641.3100.3861.4070.4668 | Ic |  |  |
| SRR7221283 | PT 21/28 | No | Yes | Yes | Yes | stx2a | 4.4.1691.3189.4019.4250.4904 | Ic |  |  |
| SRR7163893 | PT 21/28 | No | Yes | Yes | Yes | stx2a stx2c | 4.4.1691.3189.4019.4250.4946 | Ic |  |  |
| SRR6052993 | PT 21/28 | No | Yes | Yes | Yes | stx2a stx2c | 4.4.4.1215.1459.4020.4597 | Ic |  |  |
| SRR6053037 | PT 21/28 | No | Yes | Yes | Yes | stx2a stx2c | 4.4.4.1215.1459.4020.4597 | Ic |  |  |
| SRR6079443 | PT 21/28 | No | Yes | Yes | Yes | stx2a stx2c | 4.4.4.1215.1459.4020.4597 | Ic |  |  |
| SRR6079448 | PT 21/28 | No | Yes | Yes | Yes | stx2a stx2c | 4.4.4.1215.1459.4020.4597 | Ic |  |  |
| SRR7191643 | PT 21/28 | No | Yes | Yes | Yes | stx2a stx2c | 4.4.4.1215.1459.4020.4597 | Ic |  |  |
| SRR7221123 | PT 21/28 | No | Yes | Yes | Yes | stx2a stx2c | 4.4.4.1215.1459.4020.4597 | Ic |  |  |
| SRR7249522 | PT 21/28 | No | Yes | Yes | Yes | stx2a stx2c | 4.4.4.1215.1459.4020.4597 | Ic |  |  |
| SRR6079430 | PT 21/28 | No | Yes | Yes | Yes | stx2a stx2c | 4.4.4.1215.1459.4020.4646 | Ic |  |  |
| SRR6052908 | PT 21/28 | No | Yes | Yes | Yes | stx2a stx2c | 4.4.4.1362.3734.3920.4475 | Ic |  |  |
| SRR6079434 | PT 21/28 | No | Yes | Yes | Yes | stx2c | 4.4.4.1362.3734.3920.4521 | Ic |  |  |
| SRR6052891 | PT 21/28 | No | Yes | Yes | Yes | stx2a | 4.4.4.1362.3736.3924.4479 | Ic |  |  |
| SRR7221107 | PT 21/28 | No | Yes | Yes | Yes | stx2a | 4.4.4.1362.3736.3924.4479 | Ic |  |  |
| SRR6052795 | PT 21/28 | No | Yes | Yes | Yes | stx2a stx2c | 4.4.4.1408.2784.2885.4374 | Ic |  |  |
| SRR7163889 | PT 21/28 | No | Yes | Yes | Yes | stx2a stx2c | 4.4.4.1408.2784.2885.4374 | Ic |  |  |
| SRR6052822 | PT 21/28 | No | Yes | Yes | Yes | stx2a stx2c | 4.4.4.1408.2784.2885.4570 | Ic |  |  |
| SRR6053026 | PT 21/28 | No | Yes | Yes | Yes | stx2a stx2c | 4.4.4.1408.2784.2885.4570 | Ic |  |  |
| SRR7274854 | PT 21/28 | No | Yes | Yes | Yes | stx2a stx2c | 4.4.4.1408.2784.2885.4570 | Ic |  |  |
| SRR6052851 | PT 32 | No | Yes | No | Yes | stx2a stx2c | 4.4.4.2023.2770.3936.4496 | Ic |  |  |
| SRR6053023 | PT 32 | No | Yes | No | Yes | stx2a stx2c | 4.4.4.2023.2770.3936.4496 | Ic |  |  |
| SRR7282633 | PT 32 | No | Yes | No | Yes | stx2a stx2c | 4.4.4.2023.2770.3936.4496 | Ic |  |  |
| SRR7184397 | PT 32 | No | Yes | No | Yes | stx2a stx2c | 4.4.4.2023.2770.4137.4749 | Ic |  |  |
| SRR7167518 | PT 21/28 | No | Yes | Yes | Yes | stx2a stx2c | 4.4.4.2160.3025.3884.4427 | Ic |  |  |
| SRR6079438 | PT 21/28 | No | Yes | Yes | Yes | stx2a | 4.4.4.2160.3025.3928.4487 | Ic |  |  |
| SRR6052875 | PT 21/28 | No | Yes | Yes | Yes | stx2a | 4.4.4.2241.3838.4046.4632 | Ic |  |  |
| SRR7186946 | PT 21/28 | No | Yes | Yes | Yes | stx2a stx2c | 4.4.4.2304.3250.3692.4186 | Ic |  |  |
| SRR7358049 | PT 21/28 | No | Yes | Yes | Yes | stx2a stx2c | 4.4.4.2335.3905.4117.4719 | Ic |  |  |
| SRR7236607 | PT 21/28 | No | Yes | Yes | Yes | stx2a stx2c | 4.4.4.2340.2732.3997.4568 | Ic |  |  |
| SRR6052885 | PT 21/28 | No | Yes | Yes | Yes | stx2a stx2c | 4.4.4.2340.2732.4063.4653 | Ic |  |  |
| SRR7358288 | PT 21/28 | No | Yes | Yes | Yes | stx2a stx2c | 4.4.4.2340.2732.4063.4653 | Ic |  |  |
| SRR7274814 | PT 21/28 | No | Yes | Yes | Yes | stx2a stx2c | 4.4.4.2347.3957.4177.4803 | Ic |  |  |
| SRR6052813 | PT 31 | Yes | Yes | No | Yes | stx2a stx2c | 4.4.4.2508.2972.3921.4476 | Ic |  |  |
| SRR7285471 | PT 31 | Yes | Yes | No | Yes | stx2a stx2c | 4.4.4.2508.3898.4110.4712 | Ic |  |  |
| SRR6052934 | PT 4 | No | No | Yes | No | stx2c | 4.4.4.2527.3003.3933.4492 | Ic |  |  |
| SRR6053020 | PT 4 | No | No | Yes | No | stx2c | 4.4.4.2527.3003.3933.4492 | Ic |  |  |
| SRR7187823 | PT 4 | No | No | Yes | No | stx2a stx2c | 4.4.4.2527.3003.3933.4492 | Ic |  |  |
| SRR7186952 | PT 4 | No | No | Yes | No | stx2a stx2c | 4.4.4.2527.3003.3933.4664 | Ic |  |  |
| SRR6052994 | PT 32 | No | Yes | No | Yes | stx2a | 4.4.4.2541.3031.3922.4477 | Ic |  |  |
| SRR7215939 | PT 21/28 | No | Yes | Yes | Yes | stx2a stx2c | 4.4.4.2616.2175.4199.4830 | Ic |  |  |
| SRR7277870 | PT 21/28 | No | Yes | Yes | Yes | stx2a stx2c | 4.4.4.2616.3450.4160.4785 | Ic |  |  |
| SRR7167513 | PT 21/28 | No | Yes | Yes | Yes | stx2a stx2c | 4.4.4.2822.3449.3610.4184 | Ic |  |  |
| SRR6052744 | PT 21/28 | No | Yes | Yes | Yes | stx2a stx2c | 4.4.4.2980.3668.3845.4369 | Ic |  |  |
| SRR6052788 | PT 21/28 | No | Yes | Yes | Yes | stx2a stx2c | 4.4.4.2980.3668.3845.4369 | Ic |  |  |
| SRR7215923 | PT 21/28 | No | Yes | Yes | Yes | stx2a stx2c | 4.4.4.2980.3668.3845.4369 | Ic |  |  |
| SRR7256949 | PT 21/28 | No | Yes | Yes | Yes | stx2a stx2c | 4.4.4.2980.3668.3845.4369 | Ic |  |  |
| SRR7208779 | PT 21/28 | No | Yes | Yes | Yes | stx2a stx2c | 4.4.4.2998.3705.4134.4745 | Ic |  |  |
| SRR7256958 | PT 21/28 | No | Yes | Yes | Yes | stx2a stx2c | 4.4.4.2998.3705.4134.4745 | Ic |  |  |
| SRR7274846 | PT 21/28 | No | Yes | Yes | Yes | stx2a stx2c | 4.4.4.2998.3705.4134.4888 | Ic |  |  |
| SRR6052808 | PT 21/28 | No | Yes | Yes | Yes | stx2a stx2c | 4.4.4.3008.3726.3912.4463 | Ic | OUTBREAK |  |
| SRR6052921 | PT 21/28 | No | Yes | Yes | Yes | stx2c | 4.4.4.3008.3726.3912.4463 | Ic | OUTBREAK |  |
| SRR6052930 | PT 21/28 | No | Yes | Yes | Yes | stx2a stx2c | 4.4.4.3008.3726.3912.4463 | Ic | OUTBREAK |  |
| SRR6053013 | PT 21/28 | No | Yes | Yes | Yes | stx2c | 4.4.4.3008.3726.3912.4463 | Ic | OUTBREAK |  |
| SRR7291579 | PT 21/28 | No | Yes | Yes | Yes | stx2a stx2c | 4.4.4.3130.3923.4138.4750 | Ic |  |  |
| SRR7265951 | PT 21/28 | No | Yes | Yes | Yes | stx2a stx2c | 4.4.4.3212.4050.4290.4954 | Ic |  |  |
| SRR7179946 | PT 54 | Yes | No | No | Yes | stx2a stx2c | 4.4.4.3227.4072.4314.4982 | Ic |  |  |
| SRR7208993 | PT 54 | Yes | No | No | Yes | stx2a stx2c | 4.4.4.3227.4072.4314.4985 | Ic |  |  |
| SRR7236592 | PT 54 | Yes | No | No | Yes | stx2a stx2c | 4.4.4.3227.4072.4314.4985 | Ic |  |  |
| SRR7223105 | PT 21/28 | No | Yes | Yes | Yes | stx2a | 4.4.4.590.1280.3966.4532 | Ic |  |  |
| SRR6001344 | PT 21/28 | No | Yes | Yes | Yes | stx2a stx2c | 4.4.4.590.1280.3966.4548 | Ic |  |  |
| SRR6052868 | PT 21/28 | No | Yes | Yes | Yes | stx2c | 4.4.4.590.1280.3966.4548 | Ic |  |  |
| SRR6052929 | PT 21/28 | No | Yes | Yes | Yes | stx2a stx2c | 4.4.4.590.1280.3966.4548 | Ic |  |  |
| SRR6053006 | PT 21/28 | No | Yes | Yes | Yes | stx2a stx2c | 4.4.4.590.1280.3966.4548 | Ic |  |  |
| SRR6001310 | PT 21/28 | No | Yes | Yes | Yes | stx2a stx2c | 4.4.4.590.1280.4005.4578 | Ic |  |  |
| SRR7266820 | PT 21/28 | No | Yes | Yes | Yes | stx2a stx2c | 4.4.4.590.1458.3375.4168 | Ic |  |  |
| SRR7249678 | PT 21/28 | No | Yes | Yes | Yes | stx2a stx2c | 4.4.4.590.1458.3858.4513 | Ic |  |  |
| SRR7230229 | PT 21/28 | No | Yes | Yes | Yes | stx2a stx2c | 4.4.4.590.1458.3858.4572 | Ic |  |  |
| SRR6052842 | PT 21/28 | No | Yes | Yes | Yes | stx2a | 4.4.4.590.2621.3908.4459 | Ic |  |  |
| SRR6053001 | PT 21/28 | No | Yes | Yes | Yes | stx2a | 4.4.4.590.2621.3908.4459 | Ic |  |  |
| SRR7163934 | PT 21/28 | No | Yes | Yes | Yes | stx2a | 4.4.4.590.2621.3908.4459 | Ic |  |  |
| SRR7291024 | PT 21/28 | No | Yes | Yes | Yes | stx2a | 4.4.4.590.2621.3908.4459 | Ic |  |  |
| SRR7367577 | PT 21/28 | No | Yes | Yes | Yes | stx2a | 4.4.4.590.2621.3908.4522 | Ic |  |  |
| SRR7265860 | PT 21/28 | No | Yes | Yes | Yes | stx2a | 4.4.4.590.2621.3908.4576 | Ic |  |  |
| SRR6001321 | PT 21/28 | No | Yes | Yes | Yes | stx2a | 4.4.4.590.2621.3908.4592 | Ic |  |  |
| SRR7251455 | PT 21/28 | No | Yes | Yes | Yes | stx2a | 4.4.4.590.3038.4183.4811 | Ic |  |  |
| SRR7367385 | PT 21/28 | No | Yes | Yes | Yes | stx2a | 4.4.4.590.3144.3276.4399 | Ic |  |  |
| SRR7167541 | PT 21/28 | No | Yes | Yes | Yes | stx2a | 4.4.4.590.3144.3276.4406 | Ic |  |  |
| SRR6079420 | PT 21/28 | No | Yes | Yes | Yes | stx2a | 4.4.4.590.3144.3276.4464 | Ic |  |  |
| SRR7257043 | PT 21/28 | No | Yes | Yes | Yes | stx2a | 4.4.4.590.3144.3276.4464 | Ic |  |  |
| SRR6052997 | PT 21/28 | No | Yes | Yes | Yes | stx2a | 4.4.4.590.3318.3479.4610 | Ic |  |  |
| SRR7223049 | PT 21/28 | No | Yes | Yes | Yes | stx2a | 4.4.4.590.3318.3479.4755 | Ic |  |  |
| SRR7265962 | PT 21/28 | No | Yes | Yes | Yes | stx2a | 4.4.4.590.3318.3479.4905 | Ic |  |  |
| SRR7291635 | PT 21/28 | No | Yes | Yes | Yes | stx2a stx2c | 4.4.4.590.3533.3701.4196 | Ic |  |  |
| SRR6052060 | PT 21/28 | No | Yes | Yes | Yes | stx2a stx2c | 4.4.4.590.3533.3701.4201 | Ic |  |  |
| SRR6052783 | PT 21/28 | No | Yes | Yes | Yes | stx2a | 4.4.4.590.3689.3898.4449 | Ic |  |  |
| SRR6079449 | PT 21/28 | No | Yes | Yes | Yes | stx2a | 4.4.4.590.3689.3898.4449 | Ic |  |  |
| SRR7251026 | PT 21/28 | No | Yes | Yes | Yes | stx2a | 4.4.4.590.3696.3875.4412 | Ic |  |  |
| SRR7282617 | PT 21/28 | No | Yes | Yes | Yes | stx2a | 4.4.4.590.3732.3918.4472 | Ic |  |  |
| SRR6052889 | PT 21/28 | No | Yes | Yes | Yes | stx2a | 4.4.4.590.3733.3919.4474 | Ic |  |  |
| SRR6001311 | PT 21/28 | No | Yes | Yes | Yes | stx2a | 4.4.4.590.3733.3919.4480 | Ic |  |  |
| SRR6052903 | PT 21/28 | No | Yes | Yes | Yes | stx2a | 4.4.4.590.3733.3919.4655 | Ic |  |  |
| SRR6052918 | PT 21/28 | No | Yes | Yes | Yes | stx2a stx2c | 4.4.4.590.3749.3940.4500 | Ic |  |  |
| SRR7266802 | PT 21/28 | No | Yes | Yes | Yes | stx2a stx2c | 4.4.4.590.3749.4140.4752 | Ic |  |  |
| SRR7274803 | PT 21/28 | No | Yes | Yes | Yes | stx2a stx2c | 4.4.4.590.3749.4140.4752 | Ic |  |  |
| SRR7244215 | PT 21/28 | No | Yes | Yes | Yes | stx2a | 4.4.4.590.3784.3983.4550 | Ic |  |  |
| SRR6872952 | PT 21/28 | No | Yes | Yes | Yes | stx2a | 4.4.4.590.3896.4108.4709 | Ic | OUTBREAK |  |
| SRR6872960 | PT 21/28 | No | Yes | Yes | Yes | stx2a | 4.4.4.590.3896.4108.4709 | Ic | OUTBREAK |  |
| SRR6872963 | PT 21/28 | No | Yes | Yes | Yes | stx2a | 4.4.4.590.3896.4108.4709 | Ic | OUTBREAK |  |
| SRR6872964 | PT 21/28 | No | Yes | Yes | Yes | stx2a | 4.4.4.590.3896.4108.4709 | Ic | OUTBREAK |  |
| SRR6872965 | PT 21/28 | No | Yes | Yes | Yes | stx2a | 4.4.4.590.3896.4108.4709 | Ic | OUTBREAK |  |
| SRR7249752 | PT 21/28 | No | Yes | Yes | Yes | stx2a | 4.4.4.590.3896.4108.4709 | Ic | OUTBREAK |  |
| SRR6872955 | PT 21/28 | No | Yes | Yes | Yes | stx2a | 4.4.4.590.3896.4108.4850 | Ic |  |  |
| SRR7285585 | PT 21/28 | No | Yes | Yes | Yes | stx2a | 4.4.4.590.3896.4108.4850 | Ic |  |  |
| SRR7367407 | PT 21/28 | No | Yes | Yes | Yes | stx2a | 4.4.4.590.3926.4142.4754 | Ic |  |  |
| SRR7401620 | PT 21/28 | No | Yes | Yes | Yes | stx2a | 4.4.4.590.3926.4142.4764 | Ic |  |  |
| SRR7251480 | PT 21/28 | No | Yes | Yes | Yes | stx2a | 4.4.4.590.3926.4142.4766 | Ic |  |  |
| SRR7230246 | PT 21/28 | No | Yes | Yes | Yes | stx2a | 4.4.4.590.4006.4236.4881 | Ic |  |  |
| SRR7407961 | PT 21/28 | No | Yes | Yes | Yes | stx2a | 4.4.4.590.4006.4236.4881 | Ic |  |  |
| SRR7256954 | PT 21/28 | No | Yes | Yes | Yes | stx2a | 4.4.4.590.4115.4358.5035 | Ic |  |  |
| SRR6079381 | PT 21/28 | No | Yes | Yes | Yes | stx2a stx2c | 4.4.4.590.879.3828.4344 | Ic |  |  |
| SRR6052784 | PT 21/28 | No | Yes | Yes | Yes | stx2a stx2c | 4.4.4.718.1880.3817.4331 | Ic |  |  |
| SRR6052925 | PT 21/28 | No | Yes | Yes | Yes | stx2a stx2c | 4.4.4.718.3711.3893.4444 | Ic |  |  |
| SRR6001332 | PT 21/28 | No | Yes | Yes | Yes | stx2c | 4.4.4.955.2656.3156.3524 | Ic |  |  |
| SRR7179880 | PT 21/28 | No | Yes | Yes | Yes | stx2c | 4.4.4.955.2656.3156.3524 | Ic |  |  |
| SRR7265843 | PT 21/28 | No | Yes | Yes | Yes | stx2a stx2c | 4.4.4.955.4079.4321.4991 | Ic |  |  |
| SRR7277749 | PT 21/28 | No | Yes | Yes | Yes | stx2a stx2c | 4.4.4.955.4079.4321.4991 | Ic |  |  |
| SRR7291549 | PT 4 | No | No | Yes | No | stx2a stx2c | 4.4.4.955.4092.4335.5010 | Ic |  |  |
| SRR7290921 | PT 32 | No | Yes | No | Yes | stx2c stx1a | 4.402.1553.2970.3651.3827.4343 | Ib |  |  |
| SRR6052799 | PT 31 | Yes | Yes | No | Yes | stx2a stx2c | 4.570.838.1530.3780.3978.4544 | Ic |  |  |
| SRR6052816 | PT 32 | No | Yes | No | Yes | stx2a stx2c | 4.570.838.1530.3780.3978.4555 | Ic |  |  |
| SRR7367391 | PT 32 | No | Yes | No | Yes | stx2a stx2c | 4.870.1201.2226.3751.3942.4502 | Ic |  |  |
| SRR7291041 | PT 34 | No | No | No | Yes | stx2c | 4.870.1376.3137.3932.4148.4765 | Ic |  |  |
| SRR7236681 | PT 34 | No | No | No | Yes | stx2c | 4.870.1376.3137.3932.4148.4843 | Ic |  |  |
| SRR7221212 | PT 2 | No | No | No | Yes | stx2a | 4.870.1440.3228.4073.4315.4983 | Ic |  |  |
| SRR6052936 | PT 32 | No | Yes | No | Yes | stx2a | 4.870.1554.2971.3652.3829.4346 | Ic |  |  |
| SRR7244295 | PT 32 | No | Yes | No | Yes | stx2a | 4.870.1554.2971.3983.4208.4842 | Ic |  |  |
| SRR7186913 | PT 32 | No | Yes | No | Yes | stx2a | 4.870.1556.2973.3655.4308.4976 | Ic |  |  |
| SRR7184268 | PT 32 | No | Yes | No | Yes | stx2a stx2c | 4.870.1674.3157.3969.4191.4820 | Ic |  |  |
| SRR7367537 | PT 14 | No | No | No | No | stx2a | 4.870.1695.3255.4118.4361.5038 | Ic |  |  |
| SRR6052766 | PT 32 | No | Yes | No | Yes | stx2a | 4.870.305.2459.3676.3854.4378 | Ic |  |  |
| SRR7401651 | PT 32 | No | Yes | No | Yes | stx2a | 4.870.305.2459.3676.3854.4378 | Ic |  |  |
| SRR6052765 | PT 32 | No | Yes | No | Yes | stx2a | 4.870.305.2459.3676.3854.4384 | Ic |  |  |
| SRR6053015 | PT 32 | No | Yes | No | Yes | stx2a | 4.870.305.2459.3676.3854.4439 | Ic |  |  |
| SRR7367528 | PT 32 | No | Yes | No | Yes | stx2a | 4.870.305.2459.4009.4239.4885 | Ic |  |  |
| SRR7285439 | PT 31 | Yes | Yes | No | Yes | stx2a | 4.870.305.2878.3537.3705.4204 | Ic |  |  |
| SRR6052805 | PT 32 | No | Yes | No | Yes | stx2a | 4.870.305.605.3813.4015.4591 | Ic |  |  |
| SRR7286544 | PT 32 | No | Yes | No | Yes | stx2a | 4.870.305.660.2237.2290.4773 | Ic |  |  |
| SRR7249774 | PT 32 | No | Yes | No | Yes | stx2a | 4.870.305.660.2237.2290.4779 | Ic |  |  |
| SRR6052688 | PT 32 | No | Yes | No | Yes | stx2a stx1a | 4.870.305.660.3531.3699.4194 | Ic |  |  |
| SRR6052804 | PT 31 | Yes | Yes | No | Yes | stx2a stx2c | 4.870.317.2728.3282.3437.3889 | Ic |  |  |
| SRR6052834 | PT 32 | No | Yes | No | Yes | stx2a | 4.870.335.662.3682.3860.4390 | Ic |  |  |
| SRR7221271 | PT 32 | No | Yes | No | Yes | stx2a | 4.870.335.662.3682.3860.4390 | Ic |  |  |
| SRR7367414 | PT 32 | No | Yes | No | Yes | stx2a | 4.870.335.662.3682.3860.4390 | Ic |  |  |
| SRR6052801 | PT 32 | No | Yes | No | Yes | stx2a | 4.870.335.662.3682.3860.4393 | Ic |  |  |
| SRR6053004 | PT 32 | No | Yes | No | Yes | stx2a | 4.870.335.662.3682.3860.4537 | Ic |  |  |
| SRR7215277 | PT 32 | No | Yes | No | Yes | stx2a | 4.870.335.662.3939.4157.4778 | Ic |  |  |
| SRR7291921 | PT 54 | Yes | No | No | Yes | stx2c | 46.797.1662.3142.3941.4159.4781 | IIa |  |  |
| SRR7250951 | PT 34 | No | No | No | Yes | stx2c | 5.1001.1712.3232.4080.4322.4992 | IIa |  |  |
| SRR6052916 | PT 54 | Yes | No | No | Yes | stx2c | 5.1004.1417.3083.3834.4042.4625 | IIa |  |  |
| SRR7358277 | PT 54 | Yes | No | No | Yes | stx2c | 5.1004.1417.3145.3946.4166.4792 | IIa |  |  |
| SRR7221278 | PT 34 | No | No | No | Yes | - | 5.1059.1529.2892.3552.3721.4221 | IIa |  |  |
| SRR7291578 | PT 31 | Yes | Yes | No | Yes | stx2c | 5.1104.1676.3161.3976.4198.4829 | IIa |  |  |
| SRR7274842 | PT 31 | Yes | Yes | No | Yes | stx2a stx2c | 5.1113.1699.3201.4031.4267.4925 | IIa |  |  |
| SRR7230683 | PT 31 | Yes | Yes | No | Yes | stx2a stx2c | 5.1113.1699.3201.4031.4267.4951 | IIa |  |  |
| SRR7249729 | PT 31 | Yes | Yes | No | Yes | stx2a stx2c | 5.1113.1699.3201.4031.4267.4951 | IIa |  |  |
| SRR7290971 | PT 31 | Yes | Yes | No | Yes | stx2a stx2c | 5.1113.1699.3201.4031.4267.4951 | IIa |  |  |
| SRR7291581 | PT 31 | Yes | Yes | No | Yes | stx2a stx2c | 5.1113.1699.3201.4031.4267.4951 | IIa |  |  |
| SRR7191537 | PT 34 | No | No | No | Yes | stx2a | 5.1114.1704.3216.4056.4297.4963 | IIa |  |  |
| SRR7282651 | PT 34 | No | No | No | Yes | stx2c stx1a | 5.156.1136.2214.3535.3703.4198 | IIa |  |  |
| SRR7286370 | PT 34 | No | No | No | Yes | stx2c | 5.156.1168.2761.3881.4092.4687 | IIa |  |  |
| SRR7286541 | PT 34 | No | No | No | Yes | stx2c | 5.156.1168.2761.3881.4092.4687 | IIa |  |  |
| SRR7290891 | PT 34 | No | No | No | Yes | stx2c | 5.156.1168.2761.3881.4092.4687 | IIa |  |  |
| SRR7285612 | PT 34 | No | No | No | Yes | stx2c | 5.156.1301.2450.2892.4233.4874 | IIa |  |  |
| SRR6001327 | PT 34 | No | No | No | Yes | stx2c | 5.156.1301.3006.3722.3906.4457 | IIa |  |  |
| SRR7367527 | PT 34 | No | No | No | Yes | stx2c | 5.156.1301.3136.3931.4147.4763 | IIa |  |  |
| SRR7274876 | PT 34 | No | No | No | Yes | stx2c | 5.156.1601.3175.3995.4222.4861 | IIa |  |  |
| SRR6052817 | PT 34 | No | No | No | Yes | stx2c | 5.156.1635.3087.3841.4049.4635 | IIa |  |  |
| SRR7184214 | PT 34 | No | No | No | Yes | stx2a stx2c | 5.156.1683.3174.3993.4219.4858 | IIa |  |  |
| SRR7230232 | PT 54 | Yes | No | No | Yes |  | 5.156.490.2712.4102.4345.5021 | IIa |  |  |
| SRR6052177 | PT 54 | Yes | No | No | Yes | stx2c | 5.156.490.2876.3532.3700.4195 | IIa |  |  |
| SRR6052708 | PT 54 | Yes | No | No | Yes | stx2c | 5.156.490.2876.3532.3700.4200 | IIa |  |  |
| SRR7286593 | PT 54 | Yes | No | No | Yes | stx2c | 5.156.490.2876.3532.3700.4202 | IIa |  |  |
| SRR6052998 | PT 34 | No | No | No | Yes | stx2c | 5.156.490.2879.3538.3948.4511 | IIa |  |  |
| SRR6053025 | PT 34 | No | No | No | Yes | stx2c | 5.156.490.3064.3809.4011.4586 | IIa |  |  |
| SRR7291039 | PT 32 | No | Yes | No | Yes | stx2c | 5.156.490.3064.3809.4011.4994 | IIa |  |  |
| SRR6053009 | PT 54 | Yes | No | No | Yes | stx2a stx2c | 5.156.490.3108.3872.4082.4677 | IIa |  |  |
| SRR7223072 | PT 54 | Yes | No | No | Yes | stx2c | 5.156.490.3169.3985.4210.4846 | IIa |  |  |
| SRR7184306 | PT 54 | Yes | No | No | Yes | stx2c | 5.156.490.3172.3991.4216.4855 | IIa |  |  |
| SRR7251000 | PT 54 | Yes | No | No | Yes | stx2c | 5.156.490.925.2793.3501.3941 | IIa |  |  |
| SRR6079385 | PT 34 | No | No | No | Yes | stx2c | 5.156.490.925.3658.3835.4354 | IIa |  |  |
| SRR6052935 | PT 34 | No | No | No | Yes | stx2c | 5.156.490.925.3738.3926.4484 | IIa |  |  |
| SRR7286385 | PT 34 | No | No | No | Yes |  | 5.156.490.925.3797.3996.4567 | IIa |  |  |
| SRR6052927 | PT 32 | No | Yes | No | Yes | stx2c | 5.185.1279.2375.2790.3952.4516 | IIa |  |  |
| SRR7401649 | PT 32 | No | Yes | No | Yes | stx2c | 5.185.1279.2375.2790.3952.4516 | IIa |  |  |
| SRR7204345 | PT 34 | No | No | No | Yes | stx2c | 5.185.1279.2375.2790.3952.4583 | IIa |  |  |
| SRR6001333 | PT 54 | Yes | No | No | Yes | stx2c | 5.189.1275.2368.2778.3882.4425 | IIa |  |  |
| SRR7291030 | PT 54 | Yes | No | No | Yes | stx2c | 5.189.1719.3239.4087.4329.5000 | IIa |  |  |
| SRR6052820 | PT 54 | Yes | No | No | Yes | stx2c | 5.189.483.2995.3700.3879.4421 | IIa |  |  |
| SRR7215512 | PT 54 | Yes | No | No | Yes | stx2c | 5.189.483.3251.4110.4353.5030 | IIa |  |  |
| SRR7251412 | PT 70 | No | No | Yes | Yes | stx2c | 5.194.1715.3235.4083.4325.4996 | IIa |  |  |
| SRR7186915 | PT 74 | No | No | Yes | Yes | stx2c | 5.194.1715.3235.4083.4325.4996 | IIa |  |  |
| SRR7266828 | PT 54 | Yes | No | No | Yes | stx2c | 5.201.1669.3151.3961.4182.4810 | IIa |  |  |
| SRR6052873 | PT 31 | Yes | Yes | No | Yes | stx2c | 5.302.526.3079.3829.4036.4615 | IIa |  |  |
| SRR6079444 | PT 31 | Yes | Yes | No | Yes | stx2c | 5.302.526.3079.3829.4036.4638 | IIa |  |  |
| SRR6053040 | PT 31 | Yes | Yes | No | Yes | stx2c | 5.302.526.3079.3829.4036.4656 | IIa |  |  |
| SRR6053042 | PT 91 |  |  |  |  | stx2c | 5.312.1623.3067.3816.4019.4596 | IIa |  |  |
| SRR6052894 | PT 34 | No | No | No | Yes | stx2a stx2c | 5.6.310.3073.3822.4028.4606 | IIa |  |  |
| SRR7367473 | PT 2 | No | No | No | Yes | stx2a stx2c | 5.772.1106.2058.3860.4069.4662 | IIa |  |  |
| SRR7187058 |  |  |  |  |  | stx2a stx2c | 5.772.1106.2058.3860.4069.4662 | IIa |  |  |
| SRR7265925 | PT 34 | No | No | No | Yes | stx2a stx2c | 5.772.1130.3126.3913.4126.4736 | IIa |  |  |
| SRR7184307 | PT 34 | No | No | No | Yes | stx2a stx2c | 5.772.1130.3126.3913.4126.4783 | IIa |  |  |
| SRR6052904 | PT 34 | No | No | No | Yes | stx2a stx2c | 5.772.1448.3070.3819.4024.4602 | IIa |  |  |
| SRR6052811 | PT 34 | No | No | No | Yes | stx2c | 5.772.1448.3105.3866.4075.4670 | IIa |  |  |
| SRR7186927 | PT 34 | No | No | No | Yes | stx2c | 5.772.1448.3105.3866.4075.4670 | IIa |  |  |
| SRR7265866 | PT 34 | No | No | No | Yes | stx2c | 5.772.1448.3105.3942.4161.4786 | IIa |  |  |
| SRR7223139 | PT 34 | No | No | No | Yes | stx2c | 5.772.1448.3211.4047.4285.4945 | IIa |  |  |
| SRR6052854 | PT 34 | No | No | No | Yes | stx2a stx2c | 5.772.1626.3071.3820.4026.4604 | IIa |  |  |
| SRR6052919 | PT 34 | No | No | No | Yes | stx2a stx2c | 5.772.1626.3071.3820.4026.4604 | IIa |  |  |
| SRR6053029 | PT 34 | No | No | No | Yes | stx2a stx2c | 5.772.1626.3071.3820.4026.4604 | IIa |  |  |
| SRR6052791 | PT 1 | yes | No | Yes | No | stx2c | 56.1081.1577.3016.3741.3930.4489 | Outgroup |  |  |
| SRR7251454 | PT 8 | Yes | No | No | No | stx2c | 56.1103.1666.3147.3950.4170.4796 | Outgroup |  |  |
| SRR6052874 | PT 1 | yes | No | Yes | No | stx2c | 56.475.1581.3025.3755.3949.4512 | Outgroup |  |  |
| SRR7277752 | PT 1 | yes | No | Yes | No | stx2c | 56.475.1681.3170.3988.4213.4849 | Outgroup |  |  |
| SRR7286520 | PT 14 | No | No | No | No | stx2c | 56.475.1682.3173.3992.4217.4856 | Outgroup |  |  |
| SRR7407945 | PT 54 | Yes | No | No | Yes | stx2c | 56.475.1687.3183.4010.4240.4889 | Outgroup |  |  |
| SRR7290983 | PT 2 | No | No | No | Yes | stx2a stx2c | 7.1056.1522.2874.3529.3697.4192 | I/II |  |  |
| SRR6052827 | PT 4 | No | No | Yes | No | stx2a | 7.1077.1562.2989.3687.3866.4400 | I/II |  |  |
| SRR6079435 | PT 4 | No | No | Yes | No | stx2a stx2c | 7.52.1573.3010.3729.3915.4467 | I/II |  |  |
| SRR6079436 | PT 4 | No | No | Yes | No | stx2a stx2c | 7.52.1573.3010.3729.3915.4467 | I/II |  |  |
| SRR6001331 | PT 4 | No | No | Yes | No | stx2a stx2c | 7.52.1583.3028.3759.3954.4518 | I/II |  |  |
| SRR7215914 | PT 4 | No | No | Yes | No | stx2a stx2c | 7.52.1706.3219.4060.4301.4969 | I/II |  |  |
| SRR6052749 | PT 4 | No | No | Yes | No | stx2a stx2c | 7.52.83.2889.3549.3718.4317 | I/II |  |  |
| SRR7215898 | PT 4 | No | No | Yes | No | stx2a stx2c | 7.52.83.2889.3549.3718.4317 | I/II |  |  |
| SRR7286585 | PT 4 | No | No | Yes | No | stx2a stx2c | 7.52.83.2889.3549.3718.4317 | I/II |  |  |
| SRR6001290 | PT 4 | No | No | Yes | No | stx2a stx2c | 7.52.83.2889.3549.3718.4330 | I/II |  |  |
| SRR6052695 | PT 4 | No | No | Yes | No | stx2a stx2c | 7.52.83.2955.3630.3805.4314 | I/II |  |  |
| SRR7358022 | PT 34 | No | No | No | Yes | stx2c | 74.742.1282.2381.2801.4076.4671 | IIa |  |  |
| SRR7286546 | PT 34 | No | No | No | Yes | stx2c | 74.742.1282.3141.3940.4158.4780 | IIa |  |  |
| SRR6052771 | PT 54 | Yes | No | No | Yes |  | 8.1043.1561.2988.3685.3863.4397 | IIa |  |  |
| SRR7249507 | PT 34 | No | No | No | Yes | stx2c | 8.1116.1713.3233.4081.4323.4993 | IIa |  |  |
| SRR7286555 | PT 14 | No | No | No | No | stx2c | 82.861.1693.3193.4023.4254.4909 | Ia |  |  |
| SRR6053024 | PT 2 | No | No | No | Yes | stx2a | 9.146.295.2984.3678.3856.4383 | I/II |  |  |
| SRR6324154 | PT 2 | No | No | No | Yes | stx2a stx2c | 9.148.298.1994.2265.4266.4924 | I/II |  |  |
| SRR7186958 | PT 2 | No | No | No | Yes | stx2a | 9.148.298.681.3631.3806.4315 | I/II |  |  |
| SRR7283830 | PT 2 | No | No | No | Yes | stx2a | 9.148.298.681.3631.3806.4315 | I/II |  |  |
| SRR6324132 | PT 34 | No | No | No | Yes | stx2a | 9.148.298.681.3937.4155.4776 | I/II |  |  |
| SRR6324133 | PT 2 | No | No | No | Yes | stx2a | 9.148.298.681.4005.4232.4873 | I/II | OUTBREAK |  |
| SRR6324134 | PT 2 | No | No | No | Yes | stx2a | 9.148.298.681.4005.4232.4873 | I/II | OUTBREAK |  |
| SRR6324138 | PT 2 | No | No | No | Yes | stx2a | 9.148.298.681.4005.4232.4873 | I/II | OUTBREAK |  |
| SRR6324143 | PT 2 | No | No | No | Yes | stx2a | 9.148.298.681.4005.4232.4873 | I/II | OUTBREAK |  |
| SRR6324144 | PT 2 | No | No | No | Yes | stx2a | 9.148.298.681.4005.4232.4873 | I/II | OUTBREAK |  |
| SRR6324147 | PT 2 | No | No | No | Yes | stx2a | 9.148.298.681.4005.4232.4873 | I/II | OUTBREAK |  |
| SRR6324151 | PT 2 | No | No | No | Yes | stx2a | 9.148.298.681.4005.4232.4873 | I/II | OUTBREAK |  |
| SRR6324156 | PT 2 | No | No | No | Yes | stx2a | 9.148.298.681.4005.4232.4873 | I/II | OUTBREAK |  |
| SRR6324267 | PT 2 | No | No | No | Yes | stx2a | 9.148.298.681.4005.4232.4873 | I/II | OUTBREAK |  |
| SRR6324285 | PT 2 | No | No | No | Yes | stx2a | 9.148.298.681.4005.4232.4873 | I/II | OUTBREAK |  |
| SRR7256997 | PT 2 | No | No | No | Yes | stx2a | 9.148.298.681.4005.4232.4873 | I/II | OUTBREAK |  |
| SRR6324150 | PT 2 | No | No | No | Yes | stx2a | 9.148.298.681.4005.4232.4887 | I/II |  |  |
| SRR6324274 | PT 2 | No | No | No | Yes | stx2a | 9.148.298.681.4005.4232.4919 | I/II |  |  |
| SRR6324183 | PT 2 | No | No | No | Yes | stx2a | 9.148.298.681.4005.4232.4930 | I/II |  |  |
| SRR6324149 | RDNC |  |  |  |  | stx2a | 9.165.320.3134.3928.4144.4758 | I/II |  |  |
| SRR6324145 | RDNC |  |  |  |  | stx2a | 9.165.320.3134.3928.4144.4774 | I/II |  |  |
